# Liquid–liquid phase separation and the formation of amyloid fibrils from DcpS scavenger enzymes

**DOI:** 10.1038/s41598-026-55504-x

**Published:** 2026-05-27

**Authors:** Aleksandra Ferenc-Mrozek, Maria Winiewska-Szajewska, Hanna Nieznańska, Anna Anielska-Mazur, Marek Warzecha, Wojciech Dzwolak, Maciej Łukaszewicz

**Affiliations:** 1 Division of Biophysics, Faculty of Physics, University of Warsaw, Pasteura 5, 02-093 Warsaw, Poland; 2 Institute of Biochemistry and Biophysics PAS, Pawinskiego 5a, 02-106 Warsaw, Poland; 3 Laboratory of Electron Microscopy, Nencki Institute of Experimental Biology PAS, Pasteura 3, 02-093 Warsaw, Poland; 4 Faculty of Chemistry, Biological and Chemical Research Centre, University of Warsaw, Pasteura 1, 02-093 Warsaw, Poland

**Keywords:** DcpS, Decapping, LLPS, Amyloid fibrils, Biochemistry, Biological techniques, Biophysics, Cell biology

## Abstract

Decapping Scavenger (DcpS) enzyme was initially identified by its ability to hydrolyze the cap structure resulting from mRNA decay. Human DcpS is an established target for acute myeloid leukemia (AML) and hepatic metastasis. Recently, the protein has been linked to neuronal development regulation and implicated in certain developmental neurological disorders. Here we demonstrate for the first time that DcpS of the human and *C. elegans* nematode origin undergoes misfolding in vitro, leading to the formation of amyloid-like fibrils. Additionally, the DcpS^INS15^ insertional mutant linked to the Al-Raqad syndrome exhibited accelerated fibril aggregation kinetics compared to the wild type protein. Importantly, we demonstrate that the DcpS species investigated in this study undergo liquid–liquid phase separation (LLPS), which appears to lead in turn to amyloid formation. We propose that the LLPS phase transition underlies the intricate kinetics (e.g. lack of a clearly-resolved lag phase) of the misfolding process. As the physiological implications of the here-reported propensity of DcpS to lose its biological function through the coupled LLPS-fibrillization transition remain to be elucidated, this work lays the groundwork for further studies on this phenomenon and provides a potential link between DcpS aggregation and disease-associated loss of function.

**Supplementary Information:**

The online version contains supplementary material available at 10.1038/s41598-026-55504-x.

## Introduction

Most proteins have a well-defined, stable three-dimensional structure under near-physiological conditions. Stress factors like elevated temperature^1^, pressure^2^, non-physiological pH^3^ or presence of chaotropic cosolutes and cosolvents^4^ can perturb protein’s native structure. Depending on the type of perturbation the unfolding process can acquire different characteristics^5^. Destabilized proteins can aggregate either directly from the native state or via a transient intermediate states^6^. Those fully or partially unfolded intermediates (e.g. molten-globule-like states^7^) serve as aggregation precursors^8^. Many proteins contain short hydrophobic sequence segments (5–15 residues), known as aggregation-prone regions (APRs)^9^. In the native structure, APRs are typically buried within the hydrophobic core but, under unfolding conditions, the likelihood of their exposure to the solvent increases, facilitating self-association and triggering aggregation^10^. Aggregation is a highly complex process, yielding products varying in morphology, ranging from soluble oligomers to insoluble amorphous aggregates and amyloid fibrils^11^. A characteristic feature of amyloid aggregates is their hierarchical structural organization, wherein fibrils are formed through the lateral association of protofilaments assembled from stacked β-sheets^12^. The formation of amyloid fibrils is closely associated with both biological functions (e.g. formation of functional amyloids) and human diseases^13^.

Amyloid aggregation is a hallmark of numerous degenerative disorders, including Alzheimer’s disease (AD) and other tauopathies, Parkinson’s disease, type II diabetes^14^, and systemic amyloidosis e.g. immunoglobulin light chain (AL) amyloidosis^15^. In humans, more than 60 proteins have been identified in their amyloid, disease-associated forms, as reviewed recently^16^. Several mechanisms of amyloid formation have been proposed^17^, with the most widely accepted being the nucleation-dependent polymerization model, characterized by a sigmoidal aggregation kinetic curve. This model proposes that amyloid fibril formation occurs through an initial lag phase, followed by elongation of fibrils^18–21^. Other mechanisms (e.g. nucleation-independent^22^) have also been proposed. In recent years, increasing attention has been given to the role of liquid–liquid phase separation (LLPS) in pathological protein aggregation. LLPS is an intriguing phenomenon observed in cellular environments, leading to the formation of membraneless organelles or biomolecular condensates involved in various cellular processes^23–25^, including signaling, transcription^26^, RNA processing and protection under stress conditions^27^. While the formation of droplets via the LLPS pathway is typically reversible, if proteins within the droplets are subjected to persistent stress conditions, or if the LLPS-induced increased protein concentration is coupled with structure-destabilizing mutations in the protein sequence, the occurrence of droplets may facilitate formation of insoluble aggregates within^28–30^. Emerging evidence suggests that some amyloidogenic proteins implicated in various diseases^31,32^, including neurodegenerative disorders such as amyotrophic lateral sclerosis (ALS) and frontotemporal dementia (FTD)^33^ or cancer^34^, can form droplets prior to amyloid fibrils.

Here, we investigated the propensity of the decapping scavenger enzyme (DcpS) to form fibrillar aggregates under thermal stress conditions and at the physiological temperature (37 °C). DcpS belongs to the superfamily of HIT (Histidine Triad) proteins, which includes hydrolases and nucleotide transferases^35,36^. DcpS proteins are found in eukaryotes of varying complexity, including humans, nematodes and yeast^37,38^. Screening experiments have confirmed that the gene encoding human DcpS is one of the factors that determine cell viability^39^. The primary recognized function of DcpS is its role in mRNA turnover^40^. It hydrolyzes dinucleotide cap structures or short capped oligonucleotides resulting from the processive exosome mRNA degradation in the 3’ to 5’ direction^37,41^. Independent of its catalytic activity, DcpS also functions as a transcript-specific modulator^42^ involved in the pre-mRNA splicing^43^ and regulation of miRNA turnover^44^. Importantly, potential relevance of DcpS in certain clinical conditions has been reported. Human DcpS has been considered as a diagnostic biomarker associated with the colorectal cancer^45^ and as a therapeutic target for hepatic metastasis in uveal melanoma (UM), in which the RG3039 inhibitor of DcpS revealed diminished hepatic metastasis of UM cells in vivo in mouse model^46^, and for acute myeloid leukemia (AML) where the RG3039 exhibited anti-leukemic effects in a human AML xenograft model^47^.

Since 2015, several mutations in the *DcpS* gene have been linked to certain intellectual disabilities and thereafter classified as Al-Raqad Syndrome (ARS)^48,49^. ARS is a rare autosomal recessive disorder, first described in three descendants of a Jordanian family^49^ and in three members of a Pakistani family^48^, and later reported in a patient from an Italian family^50^ and, more recently, in a Japanese patient^51^. The clinical phenotype of ARS is associated with developmental delay, intellectual disability, as well as with minor deformations within the craniofacial region and accompanying anomalies affecting the heart, joints, and muscles^49,50^. Magnetic resonance imaging of a patient’s brain with ARS phenotype has revealed progressive demyelination of the central and peripheral nervous system^52^. Additionally, studies on cells from patients with DcpS enzyme dysfunction (harboring a homozygous c.636 + 1G > A mutation that produces the decapping-deficient mutant DcpS protein with a 15 amino acid insertion after Q212 residue) have shown impaired differentiation and growth of neurites^53^. This highlights the crucial role of DcpS activity in neuronal development and suggests that impaired mRNA metabolism may be the underlying cause of neurological diseases. It appears that diverse physical and intellectual anomalies in individuals with ARS are determined by various types and locations of mutations in DcpS^50^.

DcpS mutations, including those identified in clinical contexts (e.g. ARS), may give rise to variants exhibiting intrinsic instability and an increased tendency for aggregation. The wild-type DcpS protein may also display similar properties. However, information on the stability and aggregation propensity of DcpS proteins in aqueous solutions is limited, except for the melting temperature (T_m_) for human and nematode DcpS, obtained via dye-based differential scanning fluorimetry (DSF)^54^. Here, we conducted a more detailed study using biophysical methods to investigate the structural changes induced by thermal stress on human and *C. elegans* DcpS (CeDcpS). Using far-UV circular dichroism spectroscopy (CD), we found that both proteins undergo irreversible aggregation, forming β-sheet-rich aggregates with rising temperature. These aggregates were further examined using Fourier-transform infrared spectroscopy (FT-IR) and transmission electron microscopy (TEM), confirming their morphology and structural features characteristic of amyloid fibrils. To monitor real-time aggregation at human physiological temperature, we employed a Thioflavin T (ThT) binding assay. Our study also takes into account for the first time a mutated form of human DcpS, carrying the 15 amino acid insertion IKVSGWNVLISGHPA (DcpS^INS15^). Functional studies have demonstrated that the enzymatic activity of DcpS in patient-derived samples was significantly reduced, confirming the pathogenic nature of this mutation, which manifests in mental and physical disabilities^48^. Notably, the absence of a lag phase in fibril growth and the lack of a seeding were intriguing observations. We propose that this phenomenon may be explained by the occurrence of liquid–liquid phase separation, which is demonstrated here for DcpS.

## Results

### Aggregation prone regions of DcpS proteins

**Fig. 1:**
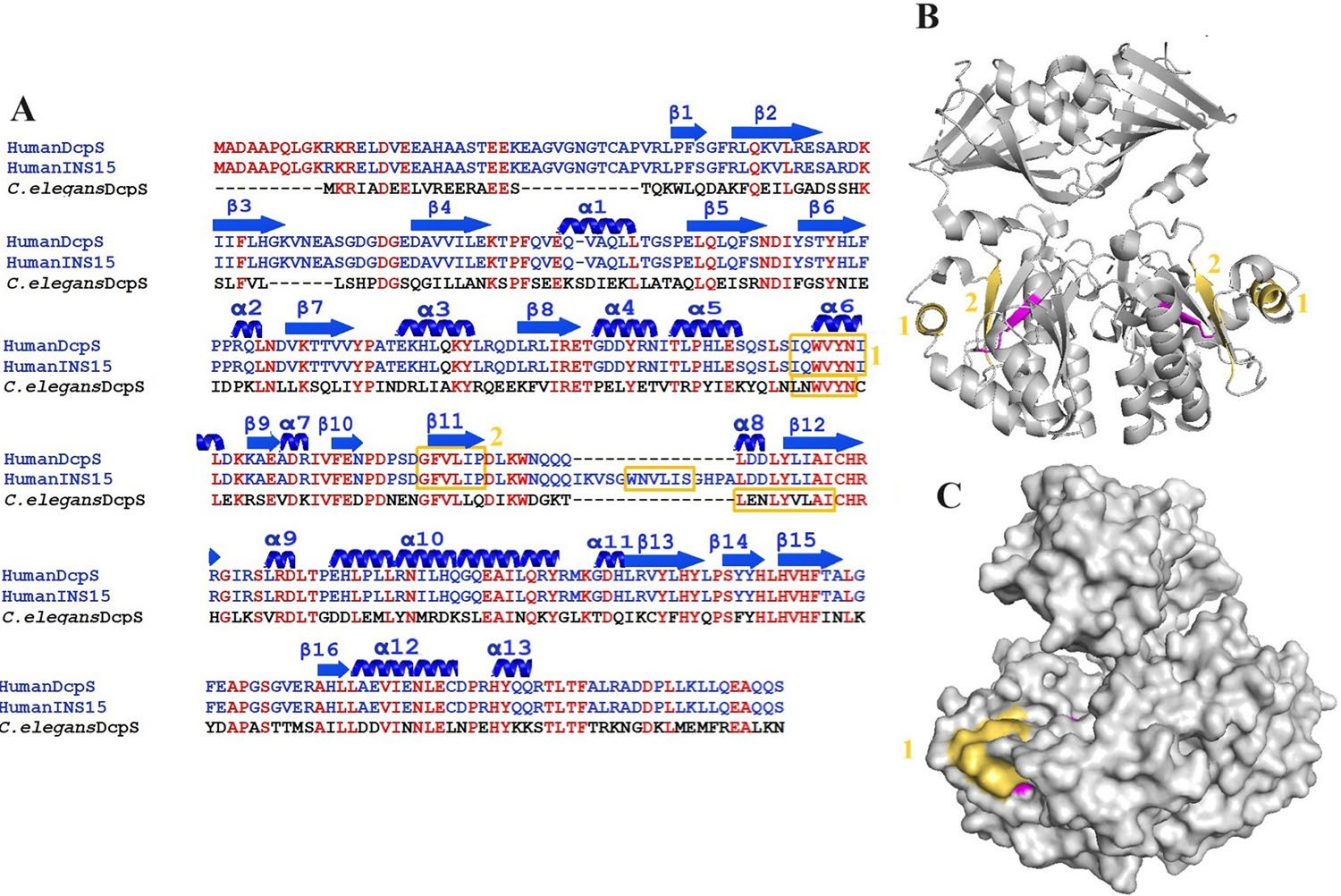
Prediction of aggregation-prone regions in DcpS proteins. (**A) **Alignment of human DcpS, mutant human DcpS (Human^INS15^) and *C. elegans* DcpS, with secondary structures location (α-helix and β-sheet) for known crystal structure of human DcpS. Amino acids labeled in red in the sequence are identical for all three proteins. APR segments are highlighted with a yellow box. *C. elegans* DcpS and human DcpS possess two APRs, while the mutant human protein has an additional region within the insertion of 15 amino acids. (**B) (C)** Crystal structure of human DcpS (PDB:1XML) with APR segments colored by yellow and catalytic centers (HIT motif) colored by magenta. Structural images were prepared using PyMOL.

Initially, we employed the WALTZ algorithm^55^ to identify putative amyloidogenic sequences in DcpS (Fig. 1, Supplementary Table 1). For the human native DcpS enzyme, WALTZ identified two potentially amyloidogenic amino acid regions within the C-terminal domain of the protein: ^173^IQWVYNI^179^ (segment no.1) and ^199^GFVLIP^204^ (segment no.2). The mutant human protein (DcpS^INS15^) has an additional region compared to the unmutated form, located within the 15-amino acid insertion: ^218^WNVLIS^223^*. C. elegans* DcpS homologue of human protein (which shares 41% of the amino acid sequence identity with human DcpS) appears to have also two segments in its sequence that can potentially contribute to amyloid formation, likewise located within C-terminal domain: ^147^LNWVYN^152^ (segment no.1) and ^187^LENLYVLAI^195^ (segment no.2). The segment no.1 overlaps in the amino acid sequence alignment of both proteins and is located on the surface of the resolved human DcpS structure (Fig. 1C), and on the surface of the modeled *C. elegans* DcpS structure (Supplementary Fig. 1B).

### Thermal denaturation of DcpS results in β-sheet aggregates

**Fig. 2:**
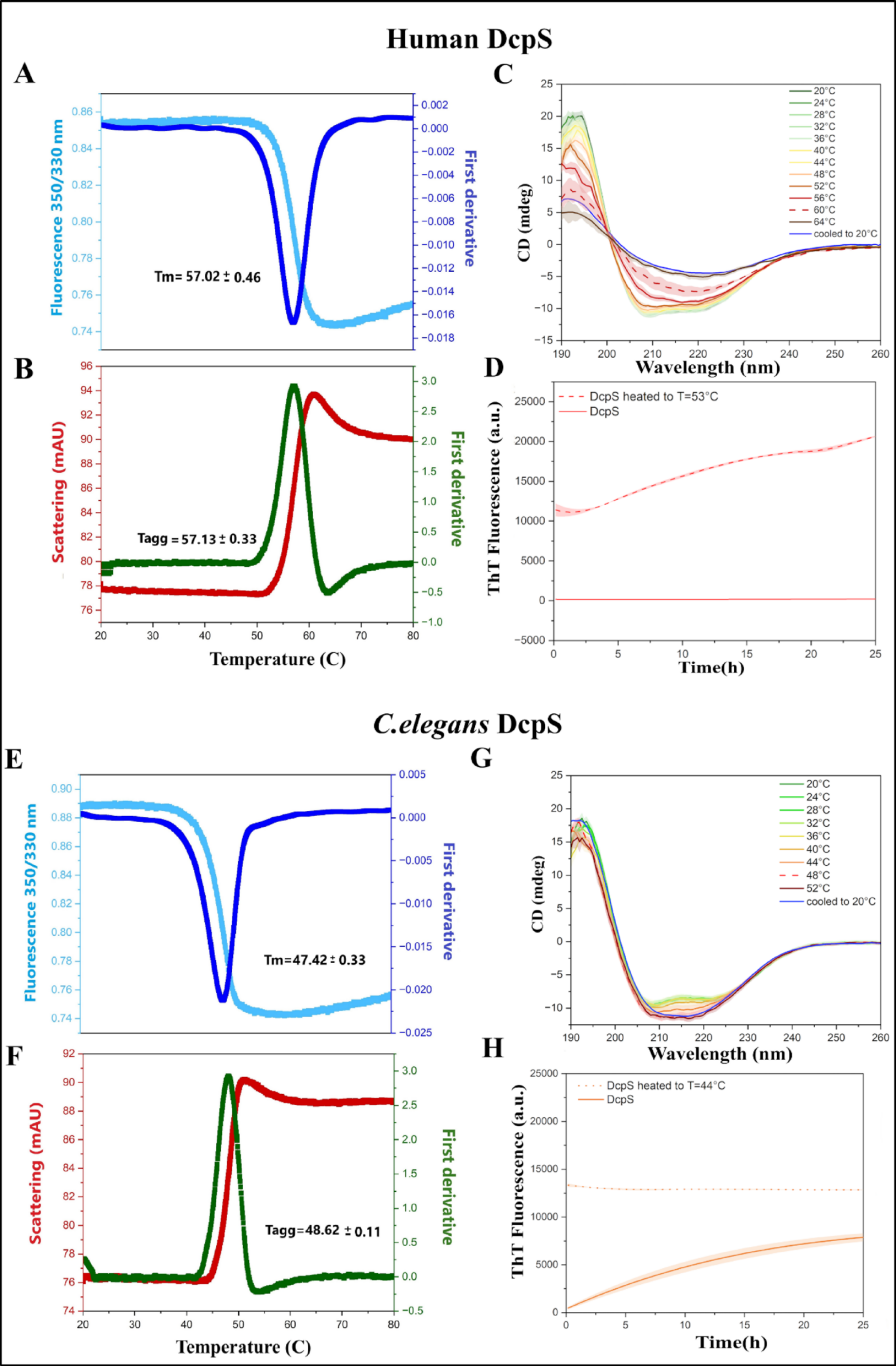
Thermal denaturation of human and *C. elegans* DcpS monitored by nanoDSF, DLS, and far-UV CD spectroscopy. **(A, E)** Comparison of thermal stability of DcpS proteins by nanoDSF in temperature range 20–80 °C. Representative thermogram trajectories correspond to fluorescence intensity ratio (350 nm/330 nm)—light blue, and it first derivative—dark blue. The temperature at which melting transition (T_m_) occurs is indicated. (**B, F)** Representative scattering profile (red) in temperature range 20–80 °C. Calculated first derivative (green) corresponds to aggregation temperature. (**C, G) **Overlay of average of smoothed far-UV CD spectra and standard deviations (colored area) obtained at temperatures ranging from 20 to 64 °C (for human DcpS) or to 52 °C (for *C. elegans* DcpS) that is 4 °C above the T_m_ temperature derived from nanoDSF (dotted line). In each case, green line is for the far-UV CD spectrum obtained at 20 °C and the series of rainbow-colored lines are spectra plots obtained at 2 °C intervals. Arrows indicate the direction of spectra development. In dark blue shown are the spectra obtained after cooling at 20 °C for 12 h of samples of human DcpS and *C. elegans* DcpS heated to 56 °C and 44 °C, respectively. (**D, H) **Aggregation kinetics of *C. elegans* and human DcpS monitored by ThT assay. Dotted lines show results obtained after heating up the DcpS sample to temperature 4 °C below the nanoDSF-derived T_m_, followed by incubation with ThT at 25 °C. The solid lines represent the fluorescence signal obtained for proteins not exposed to the raised temperature prior to the start of the ThT assay. Results presented are for a protein concentration of 0.5 mg/ml and are averaged from 3 experiments with standard deviations (colored area).

Temperature-denaturated proteins often form β-sheet-rich amyloid-like aggregates^56,57^. Initially, we decided to simultaneously monitor the thermal unfolding transition of DcpS proteins and aggregation in real-time using nano-differential scanning fluorimetry (nanoDSF) and dynamic light scattering. This approach relies on the change in intrinsic tryptophan fluorescence and aggregation-induced scattering of light by particles^58^. In Fig. 2**,** the fluorescence intensity ratio at 350 and 330 nm as a function of temperature (Fig. 2A, E) is presented, accompanied by the light scattering thermograms (Fig. 2B, F). The values obtained for the folding state transition and aggregation temperatures were nearly identical for the particular DcpS protein: T_m_ ~ 57 °C and T_agg_ ~ 57 °C for human DcpS, and T_m_ ~ 47 °C and T_agg_ ~ 48 °C for *C. elegans* DcpS, (which is in the range of the previously reported T_m_ values based on DSF with SYPRO dye^54^). Reported analysis of the charge profiles of *C. elegans* and human DcpS revealed four regions in which the two enzymes differ significantly^59^. Three of these segments are more negatively charged in CeDcpS than in hDcpS, while the fourth, located at the C-terminus, is more positively charged. All four regions are located at the dimer interface or between domains and are likely to be involved in dimerization, thus may contribute to the shown here difference in thermal stability between human and *C. elegans* DcpS. The aggregation propensity of DcpS in response to increasing temperature was further confirmed by differential scanning calorimetry (DSC), where the resulting thermograms showed both endo- and exothermic effects (Supplementary Fig. 2). Protein denaturation is generally an endothermic process, while exothermic effects typically indicate protein aggregation in solution. Because of its irreversibility, the aggregation that occurs during the protein unfolding precludes deriving quantitative thermodynamic data from such a process^60^.

In order to gain a more detailed insight into the structural transition between the folded and unfolded states, as well as the aggregation process, we recorded far-UV CD spectra in the temperature range from 20 to 64 °C for human DcpS and from 20 to 52 °C for *C. elegans* DcpS, with a gradual increase at 1 °C/2 min intervals (Fig. 2C, G). At 20 °C, the CD spectra of both DcpS proteins exhibited two distinct minima at 208 nm and 222 nm, indicative of α- helix secondary structure. The ellipticity for both the studied DcpS species vanished at temperature near the apparent T_m_ (dashed lines) (Fig. 2C, G), followed by further changes in measured CD signal. Analysis of protein secondary structure content as a function of temperature based on CD measurements (using the BeStSel server)^61^ is summarized in Supplementary Fig. 3. For human DcpS, the ratio of secondary structures remained constant up to 40 °C. Above this temperature, the β-sheet content gradually increased while the α-helical content decreased (Supplementary Fig. 3A). Noticeable changes in the secondary structure composition were observed around the melting temperature, as assessed using nanoDSF: from 56 °C onwards, the β-sheet content began to dominate over α-helices, as reflected in the corresponding CD spectra, which showed a gradual decrease in ellipticity at 208 nm and 222 nm, with the minimum at 217 nm, characteristic of β-sheet structures, becoming gradually more pronounced above 56 °C (Fig. 2C). In contrast, CD spectra of *C. elegans* DcpS showed a gradual decrease in the CD signal at 208 nm and 222 nm (Fig. 2G), suggesting either a conformational changes of protein or secondary structure rearrangement as a function of temperature. The calculated secondary structure content remained unchanged up to 44 °C, but above this temperature point the β-sheet fraction increased significantly (Supplementary Fig. 3B). It is important to note that protein aggregation and subsequent sedimentation of aggregates may lead to a reduction in the actual concentration of protein in the optical pathway during a CD measurement. The secondary structure contents were calculated, however, under the assumption of an unchanged protein concentration. Attempts to refold the proteins by cooling to 20 °C over a 12-h period (following the heating to 56 °C for human DcpS or 44 °C for *C. elegans* DcpS—so temperatures where changes in secondary structure begin to occur but before the denaturation and aggregation) were unsuccessful. As cooling of once heated samples is not accompanied by a reversal of temperature-induced changes in the far-UV CD spectra, indicating that thermal denaturation of *C. elegans* and human DcpS is irreversible.

The heat-induced aggregation of human DcpS and CeDcpS was further investigated using a ThT fluorescence assay. Upon binding to stacked β-sheets, as present in amyloid fibrils, the quantum yield of ThT fluorescence increases by 2–3 orders of magnitude which is the basis of the dye’s diagnostic applications^62^. In Fig. 2D and H, ThT fluorescence intensity was monitored for 24 h at room temperature following pre-heating of protein samples (at the heating rate of 1 °C/2 min), to the onset temperature of scattering curves (44 °C for *C. elegans* DcpS and 53 °C for human DcpS), Fig. 2B, F. Surprisingly, the initial ThT fluorescence of the partially denatured samples was significantly higher (by at least 20-fold) compared to the non-heated DcpS control. For human DcpS, ThT fluorescence further increased during incubation, whereas for *C. elegans* DcpS, it remained constant. This outcome suggests that DcpS proteins form ThT-positive aggregates, consistent with the findings from far-UV CD analysis.

### Visualization and analysis of the self-assembly of DcpS-derived fibrils

**Fig. 3:**
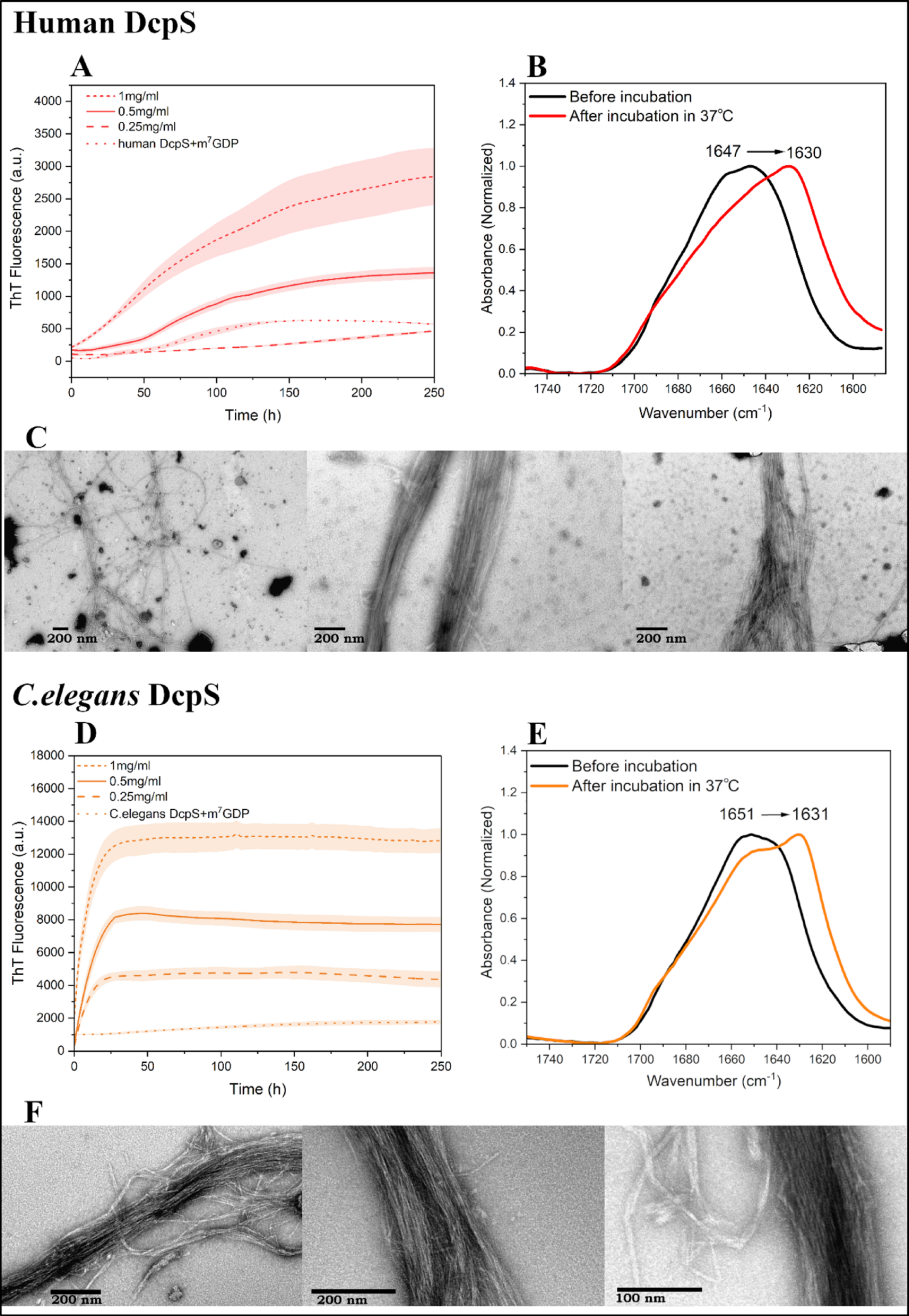
Amyloid-like fibril formation by human and *C. elegans* DcpS proteins. (**A D)** Kinetics of DcpS protein fibril formation probed by ThT fluorescence. Experiments were performed in triplicate, for indicated DcpS concentrations: 0.25 mg/ml (dashed line), 0.5 mg/ml (solid line) and 1 mg/ml (dotted line). Standard deviations are presented as colored area. Data of ThT assay in the presence of m^7^GDP at molar ratio 1:20 (protein:ligand), and at 0.5 mg/ml DcpS concentration, are presented as short dotted line. (**C, F)** TEM images of DcpS fibrillar aggregates, acquired after 10 days of incubation at 37 °C. In the case of *C. elegans* DcpS, samples were sonicated before uranyl acetate staining and TEM imaging. Scale bares are indicated on images. Three representative micrographs are shown for each protein. (**B, E)** FT-IR spectra acquired for human and *C. elegans* DcpS samples before and after 10 days incubation at 37 °C. Protein concentration at the beginning of experiment was 0.5 mg/ml.

The aggregation kinetics of DcpS were further analyzed at a constant temperature over an extended period (up to ~ 250 h). A temperature of 37 °C (human physiological temperature) was chosen, which is lower than the T_m_ and T_agg_ for both explored DcpS. While *C. elegans* nematode naturally functions at lower temperatures (20–30 °C), we opted to standardize the experimental conditions by maintaining the uniform incubation temperature. As shown in Fig. 3A and D, the intensity of ThT fluorescence for DcpS proteins increased over incubation time and was strongly dependent on the used protein concentration (similarly to e.g. insulin^63^, amyloid-β^64^ or light chains of immunoglobulin^65^—known for amyloids formation—where the ThT fluorescence intensity and the amount of formed amyloid fibrils correlates with the applied initial protein concentration). For CeDcpS, the ThT fluorescence reached plateau within approximately 24 h, with a *T*_*50*_ value (that corresponds to the time required to reach 50% of the ThT maximum fluorescence intensity in a kinetic assay^66^) of ~ 11–12 h, independent of protein concentration. For human DcpS, the maximum ThT signal was reached after 250 h or more, with *T*_*50*_ ≥ 100 h (at 1 mg/ml and 0.5 mg/ml concentrations). The curves obtained showing the kinetics of human and nematode DcpS aggregation at 37 °C point to the absence of a detectable lag phase, regardless of the protein concentration.

Tendency to amyloid aggregation can be limited by selective binding of ligands, thereby inhibiting the global process of unfolding under native or stressful conditions^67^. The m^7^GDP nucleotide is a well-known ligand that binds strongly to DcpS proteins^68^ and that it leads to the thermal stabilization of DcpS in vitro^54^. To investigate whether m^7^GDP could inhibit DcpS aggregation, we performed ThT assay in its presence. (Fig. 3A and D). At a protein-to-ligand ratio of 1:20, the ThT fluorescence intensity for both human and nematode DcpS was reduced approximately 5–sixfold compared to the sample without the ligand. Furthermore, the signal remains relatively stable even after nearly 250 h of incubation, confirming that m^7^GDP binding significantly impedes the DcpS aggregation process. This inhibition is likely due to reduction in conformational fluctuations upon m^7^GDP binding. As the ligand stabilizes the population of native-like protein molecules, the concentration of partly destabilized and aggregation-prone states is decreased, thereby slowing misfolding. Additionally, the aggregation-blocking action of m^7^GDP may be also explained by a ligand-controlled equilibrium between the open and closed conformations of the protein^69^. Such stabilization could hinder the exposure of ‘amyloidogenic regions’ to the solution and the formation of new intramolecular interactions absent in the native structure.

Structural changes resulting from thermal denaturation were monitored using FT-IR spectroscopy (Fig. 3B, E). Prior to incubation at 37 °C, the spectra indicated the dominance of α-helical structures, with the maximum of the amide I band at approximately 1647 cm^−1^ for hDcpS and 1651 cm^−1^ for CeDcpS. After 10 days of incubation at 37 °C, the band shifted toward the lower wavenumber range (1630 cm^−1^ for hDcpS and 1631 cm^−1^ for CeDcpS), indicating the presence of parallel β-sheet structures. Of note, very similar IR signatures are observed for various other amyloid aggregates—e.g. of insulin-derived fibrils^70^. The FT-IR approach allows for the specific monitoring of changes in the amide-I bond corresponding to alterations in the secondary structural conformations and state of the protein that could result from aggregation-associated transitions^71^.

The formation of the DcpS-derived amyloid-like fibrillar structures was visualized using TEM microscopy (Fig. 3C, F). Negative staining revealed that human DcpS protein can form fibrils which can be incorporated into thick bundles. These fibrils exhibited diameter of ~ 8–9 nm. Similarly, *C. elegans* DcpS formed large bundles composed of fibrils with the diameter of ~ 8–9 nm. The mature fibrils of DcpS appeared densely packed, hindering detailed morphological examination. To overcome this, the samples were sonicated before imaging. Notably, fibrils from both species were clearly visible and appeared predominantly linear, unbranched with noticeable bends. Additionally, oligomeric species were observed in the case of human DcpS samples.

### DcpS^INS15^ mutation accelerates aggregation compared to the wild-type

**Fig. 4:**
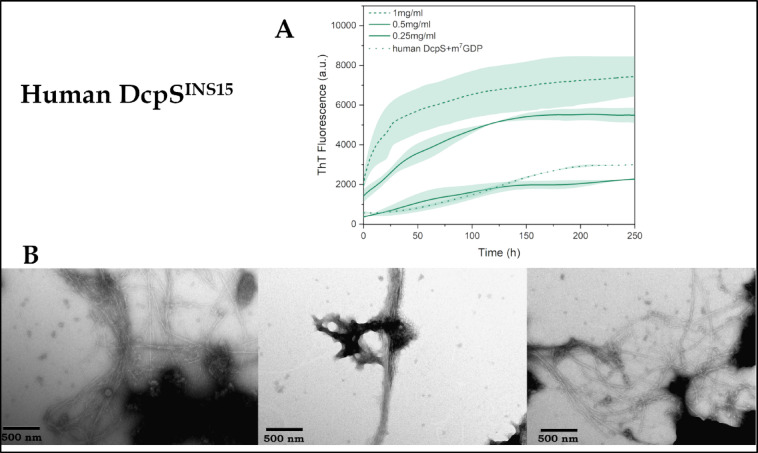
Amyloid-like fibril formation by human DcpS^INS15^ mutant protein. (**A) **Kinetics of fibril formation by human DcpS^INS15^ analyzed by ThT assay. Experiments were performed twice, for indicated DcpS concentrations: 0.25 mg/ml (short dashed line), 0.5 mg/ml (solid line) and 1 mg/ml (dashed line). No detectable lag phase is seen regardless of protein concentration. Data of ThT assay in the presence of m^7^GDP (at ligand:protein molar ratio 1:20), and at DcpS^INS15^ 0.5 mg/ml concentration, are shown as short dotted line. (**B)** TEM images of DcpS^INS15^ fibrillar aggregates acquired after 10 days of incubation at 37 °C. Samples were sonicated before uranyl acetate staining and TEM imaging. Three representative micrographs are shown. Scale bare represents 500 nm.

In parallel to wild-type hDcpS, we also analyzed aggregation propensity of DcpS^INS15^ mutant^48^ (Fig. 4). As it is shown in Supplementary Fig. 4A, the far-UV CD spectra of DcpS^INS15^ were indistinguishable from those of WT DcpS, and the protein retained its ligand binding capacity and enzymatic activity (albeit at reduced level, ~ 3x) (Supplementary Fig. 4B and 4F). The T_agg_ (53 °C) and apparent T_m_ (52 °C) for DcpS^INS15^, based on the course of the nanoDSF thermograms, were significantly lower in comparison to the values obtained for WT hDcpS (around 4 °C for T_agg_, and around 5 °C for T_m_) (Supplementary Fig. 4E). This indicates that DcpS^INS15^ has much lower thermal protein stability and is more prone to aggregation at lower temperatures, and this could be a result of a 15-residue insertion in the loop that connects the lysine and tyrosine in the DcpS active site what changes its geometry, and in addition this extended loop interfere at the DcpS dimer interface in the modeled DcpS^INS15^ structure^48^. Structural analysis based on far-UV CD spectroscopy further confirmed clear changes in the content of α-helices and β-sheets above 48 °C, where the percentage of β-sheet began to increase (Supplementary Fig. 4D). In an analogy to the unmutated form, the DcpS^INS15^ was not capable of refolding after cooling of the sample and subsequent incubation at 20 °C (Supplementary Fig. 4C). Analysis of the DcpS^INS15^ aggregation kinetics at 37 °C using ThT assay showed a rapid increase in the fluorescence intensity upon incubation. The *T*_*50*_ for DcpS^INS15^ was estimated to approximately 50 h (at 0.5 and 0.25 mg/ml initial protein concentrations) and only to around 12 h at 1 mg/ml, what is comparable to *T*_*50*_ value obtained for CeDcpS and four times faster than that of WT DcpS (*T*_*50*_ ~ 50 h) (Fig. 4A). ThT fluorescence intensity in in the presence of m^7^GDP was around 3 times weaker compared to the ligand-free sample, although around four times higher than that of wild type DcpS at the end-state, suggesting that ligand binding interferes with the aggregation process similarly to the wild-type DcpS. This clearly indicates that the mutation significantly accelerates aggregation of DcpS^INS15^. Visualization of DcpS^INS15^ amyloid-like fibrils by TEM revealed the presence of tightly associated fibril clusters and amorphous aggregates incorporating short fibrils (Fig. 4B). These fibrils were ~ 8–9 nm in diameter.

### Droplet-like protein condensates of DcpS

**Fig. 5:**
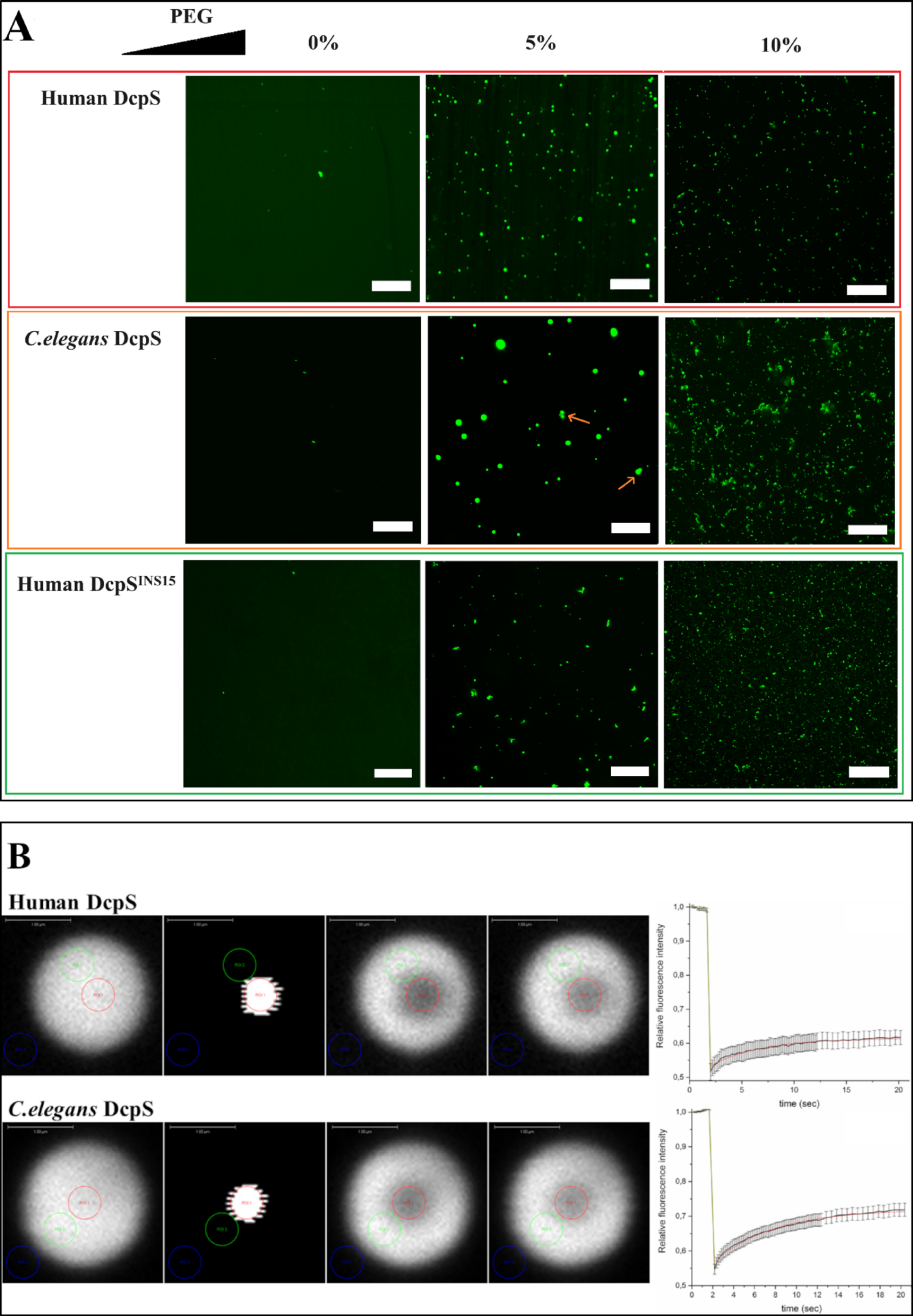
DcpS proteins undergo liquid–liquid phase separation in vitro. (**A)** Confocal microscopic images of DcpS droplets visualized with ATTO 488 fluorescent dye, at protein: dye molar ratio 1:10 (incubation at room temperature for 1.5 h). Images shows a distinct spherical droplets for *C. elegans* and human DcpS at 5% PEG-4000 crowding agent concentration. Potential fusion events between DcpS droplets are marked by red arrows. Data were obtained in 50 mM HEPES, 150 mM NaCl pH 7.5 buffer, for 1 mg/ml DcpS concentration. Scale bars correspond to 20 µm. (**B)** Representative images showing DcpS condensates during fluorescence recovery after photobleaching (FRAP): before bleaching, at bleaching, and after bleaching (0 and 20 s), accordingly from left to right. Apparent mobile fraction is around 17% and around 35% for human and *C. elegans* DcpS, respectively. The scale bar is 1 μm.

The apparent lack (or shortening) of the lag (nucleation) phase in the DcpS aggregation kinetics (Figs. 3A and D, 4A), could be rationalized as a symptom of liquid–liquid phase separation and droplet formation preceding fibrillization^72,73^. Therefore, we chose to investigate the initial stage of the aggregation process of DcpS proteins and explore the possibility that they undergo aggregation/fibrillation via LLPS mechanism. Initial screening of databases, such as LLPSDB 2.0 and PhaSePro^74,75^, did not indicate DcpS as protein known to participate in LLPS formation in vivo or in vitro. To verify experimentally the hypothesis that DcpS undergoes LLPS, we analyzed microscopic images of DcpS in the presence of 5% and 10% concentrations of polyethylene glycol (PEG-4000). PEG is used as the effective molecular crowding agent, mimicking the crowded intracellular environment, thereby facilitating the formation of protein-rich droplets^76^. As shown in the images obtained by confocal microscopy (Fig. 5A), no droplets were detected for any of the studied proteins at 0% PEG. However, at 5% PEG, distinct droplets were clearly seen, with diameters of approximately 1–3 μm for hDcpS and 2–5 μm for CeDcpS. Fluorescence recovery after photobleaching (FRAP) analysis points to the dynamic nature of the in vitro-formed condensates of DcpS proteins (Fig. 5B), however with limited fluorescence recovery (with the apparent mobile fraction of around 17–35% , and recovery half-times of 4.7 ± 0.3 and 5.8 ± 0.2 s for human and *C. elegans* DcpS, respectively). The relatively low FRAP recovery suggests the less-dynamic, partial immobility of the formed droplets, which may be due to protein aggregation within the droplets (thereby reducing the mobile fraction of DcpS molecules within the condensates). In the case of CeDcpS, examples of two droplets in very close proximity can be found among the single droplets, indicating the potential fusion of condensates (marked by arrows, Fig. 5A). For the human DcpS mutant, only single spherical droplets with a diameter of 1–2 μm were detected and, in addition, particles of variable shape were also present, suggesting protein aggregation (Fig. 5A). At 10% PEG concentration, only irregular, probably aggregated structures were observed for each of the studied DcpS.

At the right of each image panel, the corresponding time course of fluorescence recovery during FRAP experiment for human DcpS and CeDcpS (at 1:10 ratio of ATTO 488 labeled protein and unlabeled protein v/v) is shown. Data are presented as the mean + /-standard error (n = 6 independent experiments).

After 5 h of incubation at 20 °C, *C. elegans* and human DcpS droplets remained liquid-like and displayed greater dynamism, as reflected by the observation of a greater number of fusion events (Supplementary Fig. 5A). However, after a shift to 37 °C and 30 min of incubation, the CeDcpS droplets diverged into aggregate-like structures (Supplementary Fig. 5B). In the case of human DcpS, droplets appeared smaller and not perfectly spherical, suggesting little fusion between condensates (Supplementary Fig. 5B).

Visualisation of human and *C. elegans* DcpS droplets in the presence of m^7^GDP revealed protein condensates smaller in size in comparison to DcpS alone (a more pronounced effect in the case of CeDcpS), as well as the presence of irregular aggregates (Supplementary Fig. 8).Thus it seems that the m^7^GDP ligand interferes with the LLPS-droplet formation by DcpS, which may be due to changes/differences in the local distribution of charge patches on the protein surface between the closed and open forms of the DcpS dimer.

The presence of 5% PEG also had a positive effect on the aggregation kinetics of hDcpS, as the fluorescence signal of ThT started to increase earlier in comparison to the sample without PEG, with the *T*_*50*_ estimated to ~ 25 h (compared to *T*_*50*_ of PEG-free samples ~ 80–100 h) (Supplementary Fig. 6A). In the presence of 10% PEG, the ThT fluorescence remained “flat” over the incubation time, suggesting the presence of amorphic aggregates that are unable to form fibrillar structures. This suggest also that higher PEG concentrations induce structural changes in DcpS (Supplementary Fig. 6B) and higher propensity towards amorphic aggregation.

**Fig. 6:**
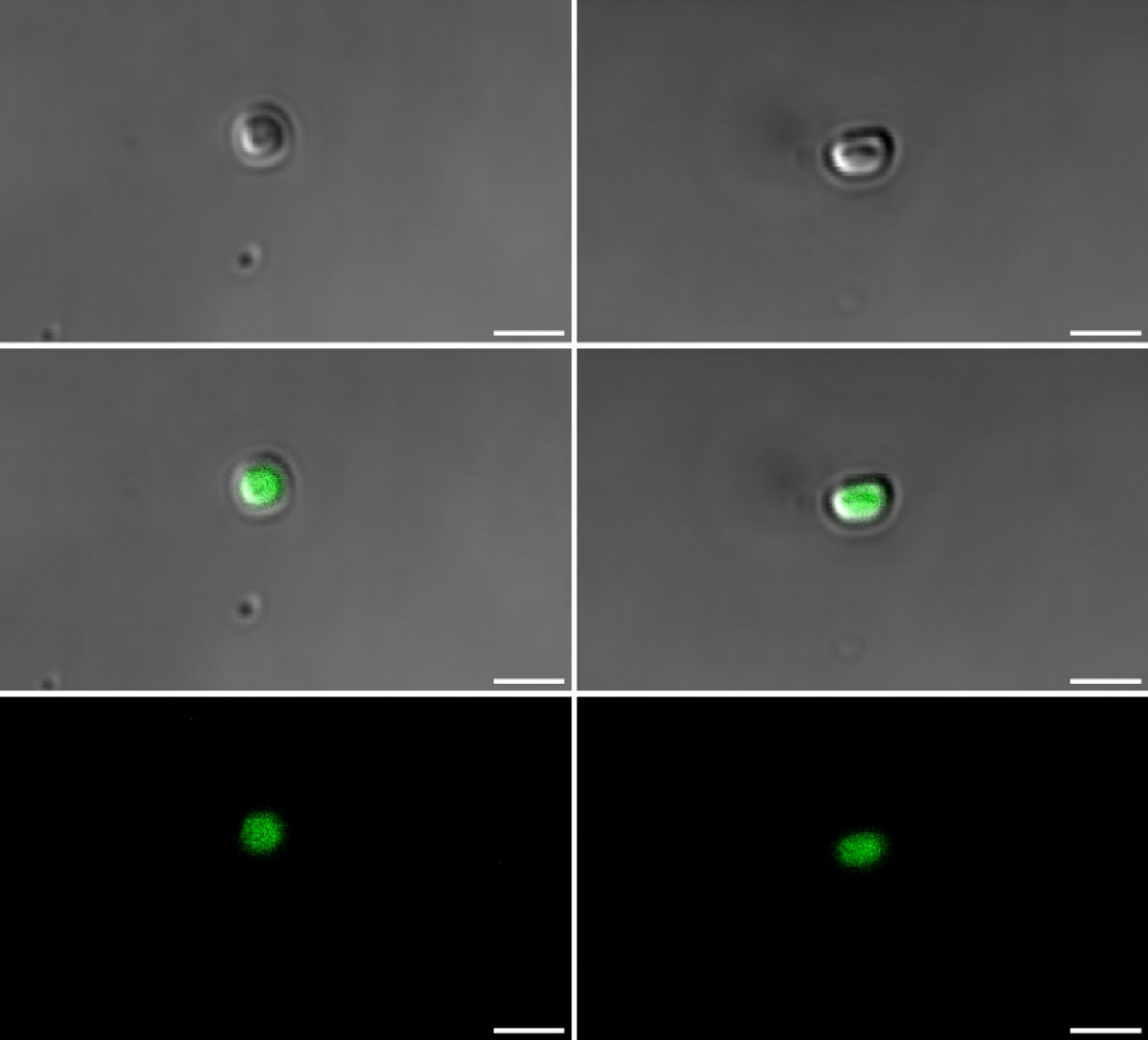
Formation of ThT-positive aggregates within DcpS droplets. Representative confocal microscopic images of *C. elegans* DcpS droplets formed in the presence PEG-4000 in a 50 mM HEPES, 150 mM NaCl, pH 7.5 buffer and 10 µM ThT. Bright-field images of CeDcpS droplets (upper panel), the corresponding merged images of ThT fluorescence and the bright-field channel (middle panel), and the ThT fluorescence alone (bottom panel) are shown. Images were acquired for samples at the start of incubation at 37 °C (t = 0 min). ThT-positive droplets can be seen amongst the formed CeDcpS droplets. Scale bar corresponds to 2 µm.

The DcpS droplets were further investigated in the presence of PEG and Thioflavin T and the juxtaposed brightfield and ThT fluorescence microscopic images are shown in Fig. 6 and in Supplementary Figs. 9A and B. The ThT-positive droplets become detectable right from the start of observation, which in turn suggests a rapid formation of amyloid aggregates within condensate (Fig. 6). After around 30 min a larger ThT-positive droplet-like aggregates are formed, irrespective of the used buffer conditions (Supplementary Figs. 9A and 9B). In the absence of PEG, no droplet-like structures were detected upon DcpS incubation with ThT up to around of 1 h (Supplementary Fig. 10A), and after around 3 h droplets and droplet-like aggregates with ThT fluorescence appeared in the analyzed samples (Supplementary Fig. 10B).

Taken together, these results demonstrate that DcpS proteins can undergo phase transition and form LLPS droplets in vitro in the artificially crowded environment, and that the formation of DcpS amyloid-like fibrils could occur in vitro via an LLPS-dependent pathway.

## Discussion

DcpS proteins have been extensively studied for their enzymatic activity in mRNA turnover, including their activity and specificity towards synthetic mRNA cap analogs—potent compounds used in designing stable therapeutic mRNA, such as mRNA vaccines, or as potential inhibitors of eIF4E—a protein overexpressed in various types of cancer^77–79^. Recently, the involvement of human DcpS and its mutants have been highlighted in the development of rare neurological disorders^48–50^ and in the regulation of neuronal development^53^, as well as in cancer diagnostics^45,46^.

The results of the present study demonstrate that DcpS can form protein amyloid-like fibrils. TEM images support such a statement, and visualized fibrillar aggregates possess morphology similar to well-known amyloid forming proteins^65,80,81^. Diameter of DcpS fibrils (~ 8–9 nm) is similar to that of well-characterized amyloid fibrils of Aβ peptides (7–12 nm)^82,83^, observed in AD. Circular dichroism spectra indicated that thermally induced aggregation is accompanied by a transition of α-helical to β-sheet structures within the temperature range examined, with the resulting structural state capable of binding Thioflavin T. Importantly, the increase in ThT fluorescence was observed at the constant temperature (37 °C), indicating the formation of amyloid-rich β-sheet aggregates by DcpS proteins from *C. elegans* and *H. sapiens* (physiological temperature for human)*.* In the context of the results obtained, we included the human DcpS^INS15^ in the study—the first clinically significant DcpS mutation revealed. DcpS^INS15^ undergoes denaturation at lower temperatures and aggregates irreversibly into amyloid structures significantly faster than the wild-type DcpS, despite the far-UV CD spectra do not reveal any significant changes in the secondary structure. From the structural point, the DcpS^INS15^ is enriched in the additional APR segment found in this additional 15-amino acid insertion, and molecular modeling revealed that this insertion is exposed to solvent^48^, what likely increases aggregate formation and decreases protein stability.

An intriguing aspect of the aggregation kinetics observed in all the DcpS species studied was the shape of the aggregation curve, which did not correspond to the classical sigmoidal pattern associated with the nucleation phase of fibril formation^20^. While factors such as temperature or salt concentration may contribute to the apparent absence of a lag phase^84^, it is also possible that rapid formation of new aggregates occurs due to pre-existing amyloid fibrils or other seeding agents^18^. They accelerate fibril growth by providing a catalytic platform for further elongation (secondary seeding), as observed for lysozyme fibrils^80^, Aβ fibrils of Alzheimer’s disease^85^, or α-Synuclein amyloid aggregates associated with Parkinson’s disease^86^. Cross-seeding—where amyloid structures of one protein serve as templates for the aggregation of another—is also a well-documented process that promotes fibril formation (e.g. in studies of the interaction between Tau and Aβ42 in Alzheimer’s disease or α-synuclein and Aβ in Parkinson’s disease^87^, or in recent reports suggesting that SARS-CoV-2 N-protein and S1 RBD protein may cross-seed α-synuclein aggregation, potentially increasing the risk of Parkinson’s disease post-transmission^88–90^. However, our results unequivocally demonstrate that fragments of sonicated mature fibrils of the wild-type hDcpS, or the DcpS^INS15^ mutant used for cross-seeding experiments, do not promote secondary nucleation or shorten the fibril growth phase of human DcpS (Supplementary Fig. 7). Whether the oligomeric species observed in TEM images for human DcpS may contribute to fibril nucleation and growth acceleration remains to be fully elucidated. Specific detection and characterization of these oligomers, their potential equilibrium with native dimeric species, and their subsequent disappearance upon fibril formation could provide valuable mechanistic insights^91^. It is important to note that some oligomeric species may have a higher tendency for dissociation to monomers than for fibril formation, as reported by Dear et. al.^92^. The apparent lack of a classical lag phase in ThT assays for DcpS does not preclude the presence of a very short lag phase that may be undetectable under the current in vitro conditions. Several mechanistic factors could explain this observation: pre-existing oligomers or transient nucleation-prone intermediates may act as seeds, accelerating fibril formation and bypassing a detectable lag phase^93^. In addition, liquid–liquid phase separation may concentrate DcpS molecules, locally increasing protein concentration and favoring rapid β-sheet formation, thereby masking the lag phase typically observed in dilute solutions^31^. Applying subtle changes in environmental conditions, such as temperature, ionic strength, or molecular crowding, one can modulate nucleation and fibril elongation kinetics, potentially influencing the length of the observable lag phase^94^.

Numerous studies showed that protein liquid–liquid phase separation could trigger the formation of protein amyloid aggregates^72,73^, and it was also proposed that LLPS could act as an alternative nucleation pathway that accelerates the aggregation^95^. In accordance with observations for other proteins that form droplets^72,96^, it is possible that the absence of a discernible lag phase for *C. elegans* DcpS, human DcpS, and its mutant is due to the existence of a LLPS mechanism. Indeed, the microscopy imaging results showed that in vitro both human and *C. elegans* DcpS proteins form droplets under molecularly crowded conditions at physiological pH and salt concentration. According to studies of other systems, two or more droplets can merge to form larger ones over time^97^. The frequency and extent of fusion events may increase during incubation time, leading to the formation of larger droplets—a phenomenon referred to as maturation^98^. Similar to the reported examples^97,98^, we observed such fusion events of LLPS droplets also for human and *C. elegans* DcpS, further supporting the notion that DcpS proteins undergo a liquid–liquid phase separation process. Furthermore, the lack of changes in aggregation rate for DcpS proteins depending on concentration can also be attributed to LLPS^96^. During the early stages of aggregation kinetics measurements, the detection of ThT fluorescence signal above zero suggests conformational changes involving β-sheet transitions in condensates^72^. This seems consistent with the detection of ThT-positive droplets at the beginning the microscopic observations.

Available studies suggest that the proteins with the intrinsically disordered regions or low complexity domains tend to have a higher propensity to form droplets^99,100^. However there are also examples of a few globular proteins undergoing liquid–liquid separation^101–103^, and now DcpS can be added to this list. The intriguing question is why DcpS proteins are not abundant in membraneless organelles like P-bodies or stress granules involved in RNA processing or mRNA degradation, similarly to other RNA-interacting proteins^27,104^. One assumption is that LLPS is known to be facilitated by long RNA molecules^105,106^, whereas DcpS processes short mRNA transcripts up to 10 nucleotides, but with the highest effectivity towards dinucleotides^41^. However, there are known droplet-forming proteins that do not interact with RNA^107,108^, therefore DcpS proteins may also have this property, which in turn could be connected to their biological functions beyond mRNA degradation (although these functions are currently unknown).

Our study has also shown that the binding of the m^7^GDP cap analog by the DcpS protein significantly limits its aggregation process. As such, m^7^GDP and other specific ligands of DcpS could be tested as potential inhibitors of DcpS-derived fibril formation or used as a starting point in designing of novel anti-aggregation compounds, similarly to the creation of compound libraries with therapeutic significance that block protein aggregation^109,110^.

Given that proteins forming amyloids in vitro do not always do so in vivo^111^, and that only about 33% of proteins that could form LLPS droplets in vitro undergo this process within the range of their cellular concentrations (with regard to the PaxDb database)^112^, a relevant question is whether DcpS could form functional (or pathological) fibrils or biomolecular condensates in vivo. Future efforts should focus on studies that address the detection of possible DcpS droplets and/or fibrils in vivo. And further, whether such DcpS droplets could then serve as, for example, an intermediate phase on the pathway to the formation of amyloid-like fibrils via creation of crowded protein molecular environment and the subsequent promotion of rapid fibril formation (what could be more pronounced for DcpS mutants, as shown here in vitro for DcpS^INS15^), or potential physiological mechanism connected to regulation of its enzymatic activity^113^. The crowded and more viscous interior of DcpS LLPS droplets may cause, for example, a transient reduction of its enzymatic activity as a consequence of reduced mobility of substrates and reaction intermediates, or alters its substrate specificity (e.g., enabling processing of longer capped oligoribonucleotide substrates next to the standard dinucleotide cap analogs). On the other hand, DcpS droplets may be involved in the regulation of miRNA turnover^44^ by sequestration of miRNA molecules. It would also be interesting whether DcpS droplets may be formed in the presence of other protein(s), similarly to another mRNA decapping enzyme, Dcp1/2^114^. Those issues require further investigation, in particular, the visualization of possible condensates in the cellular environment under different conditions. Especially, such an analysis could focus on the human mutant protein with a 15 amino acid insertion, which has shown here accelerated fibrillation and reduced ligand binding, as well as other known mutants associated with Al-Raqad syndrome^48–50,52^. DcpS substrates/ligands include m^7^GpppN and m^7^GDP nucleotides generated during mRNA degradation (via 3’-to-5’ or 5’-to-3’ pathway)^54^, thus the reduced ability of DcpS mutant(s) to bind its ligands may promote fibrillar aggregation under cellular conditions.

Impaired DcpS decapping activity has been reported for a mutant identified in ARS syndrome^48^. Further studies on iPSC cells harbouring this mutation in the DCPS gene demonstrated diminished neural differentiation and neurogenesis, and DcpS knockdown in mouse neurons negatively affected callosal axon outgrowth^53^. These findings may suggest that the formation of DcpS-derived fibrils and the resulting reduction in enzymatic activity within aggregates, or the sequestering of active molecules and lowering their intracellular levels, could produce similar detrimental effects. Future research aimed at identifying the molecular features and mechanism underlying DcpS fibrillation might help to guide the development of therapeutic strategies, for example, through the design of specific inhibitory molecules of DcpS aggregation that do not affect its enzymatic activity, or fibrill-targeting PROTACs (proteolysis-targeting chimeras)^115^. On the other hand, elucidating the molecular or metabolic pathways where DcpS activity is involved may represent another approach to overcoming the DcpS deficiency, as recently reported for the creatine biosynthesis pathway^116^.

As DcpS proteins are members of the histidine triad (HIT) superfamily of enzymes, it raises a question whether any other HIT protein has the ability to form fibrillar structures in vitro (and in vivo). Although not amyloid-like fibrils, the recent report showed that HINT1 protein from this superfamily is turned out into long polymers (from 2 to ~ 10 000 subunits) and that this is promoted by the presence of diadenosine tetraphosphate Ap4A^117^. This HINT1 polymerization is enhanced by higher concentrations of this dinucleotide, leading to filaments of ~ 10 μm in length (and 10–250 nm in width).

In conclusion, our results clearly showed that DcpS scavenger proteins, of human and *C. elegans* nematode origin, form amyloid fibrils, and it is possible that this process could proceed through the LLPS-assisted pathway. The clinically significant DcpS^INS15^ mutant is significantly less thermally stable and tends to aggregate into amyloid structures significantly faster than the wild-type DcpS.

## Methods

### Recombinant protein production and purification

Human and *C. elegans* DcpS were expressed in the *Escherichia coli* Rosetta2 (DE3) from the pET30a( +)hDcpS or pET30a( +)CeDcpS vectors, according to the procedure described previously^54^. Briefly, protein expression was induced with 0.4 mM IPTG at OD600 around 0.5–0.8, and the bacterial culture was incubated in LB medium at 18 °C overnight. The cell pellet collected by centrifugation (7000 g, 10 min) was resuspended in the ice-cold lysis buffer (50 mM TRIS pH 7.5, 300 mM NaCl, 1% Triton X-100 and 20 mM imidazole), sonicated and centrifuged at 4 °C, 30000 g, for 3 h. The supernatant was applied to a HisTrapHP column (Cytiva) equilibrated with 50 mM TRIS pH 7.5, 300 mM NaCl and the DcpS protein was eluted with an imidazole gradient (20–600 mM) with flow rate 0.5 ml/min. The final purification step was gel filtration on a Superdex 200 10/300GL column (GE Healthcare Life Sciences) using ÄKTA FPLC protein purification system (GE Healthcare Life Sciences) and appropriate (for performed later experiments) elution buffer: 50 mM TRIS pH 7.5, 150 mM NaCl, 0.2 mM TCEP, or 15 mM TRIS pH 7.5, 80 mM NaClO_4_, and 0.2 mM TCEP. The purified protein was stored at 4 °C.

Human DcpS^INS15^ was purified according to the above procedure using the pET30a + INS15hDcpS plasmid, which was generated by replacing the wild-type DcpS open reading frame with the cDNA encoding a 15-amino acid insertion^48^ at the NdeI/BamHI restriction sites of the pET30a + vector (BioCat GmbH, Germany).

### Nano-differential scanning fluorimetry (nanoDSF)

NanoDSF experiments for DcpS were performed using a NanoDSF Prometheus NT.48 device equipped with backreflection mode (NanoTemper Technologies, München, Germany). Upon measurement, capillaries were filled with a DcpS protein suspension at a final concentration of 0.5 mg/mL in 50 mM TRIS, 150 mM NaCl, 0.2 mM TCEP, pH 7.5 buffer and then subjected to thermal stress from 20 to 80 °C, with a ramp rate of 0.5 °C/min. Fluorescence emission was recorded at 330 nm and 350 nm following UV excitation at 280 nm. Protein aggregation was monitored simultaneously using back-reflection optics. All measurements were repeated 4 times. Thermal stability parameters, including T_m_ and the onset temperature of aggregation (T_agg_), were determined by implemented PR.ThermControl v2.1.2 software (NanoTemper Technologies). The results are presented as means and errors were estimated as standard deviations of four independent experiments.

### Circular dichroism (CD) spectroscopy

Circular dichroism (CD) spectra of DcpS were recorded in 15 mM TRIS, 80 mM NaClO₄, and 0.2 mM TCEP (pH 7.5) buffer with a final enzyme concentration of 0.2 mg/mL using a CD Chirascan Plus spectrometer (Applied Photophysics Ltd., Leatherhead, UK). Conformational changes in the protein secondary structure were monitored in the far-UV range between 190 and 260 nm, at 0.5 nm intervals, in a quartz cuvette with a path length of 0.5 mm. The far-UV CD spectra were recorded at temperatures range from 20 up to 64 °C (for human DcpS) or up to 52 °C (for *C. elegans* DcpS). The temperature was increased stepwise from 20 °C at a heating rate of 1 °C/0.5 min. The percentage of protein secondary structures was determined from the recorded spectra using BeStSel software^61^.

In renaturation experiments, far-UV CD spectra were recorded at 20 °C after human DcpS or *C. elegans* DcpS being heated to 56 °C or 44 °C, respectively (at a heating rate of 1 °C/0.5 min), followed by gradual cooling to 20 °C and incubation for 12 h (before measurement). All measurements were repeated 4 times, with the exception of human DcpS^INS15^ that due to aggregation problems, was performed only twice. The spectra shown are averaged from all measurements with standard deviations indicated.

### Differential scanning calorimetry (DSC)

Differential scanning calorimetry (DSC) measurements were carried out using a MicroCal VP-DSC calorimeter (Malvern Instruments, UK) in the temperature range of 20–100 °C, with a scanning rate of 0.5 °C per minute. The protein sample was prepared at a concentration of 0.7 mg/mL in a buffer containing 50 mM TRIS, 150 mM NaCl, and 0.2 mM TCEP at pH 7.5. Prior to DSC measurements, both the protein solution and the buffer used as a reference were degassed under vacuum. For each experiment, scans buffer–buffer and sample–buffer were performed under identical conditions. Final thermograms were obtained by subtracting the appropriate reference baseline (buffer–buffer) from the protein thermogram. The resulting excess heat capacity curves were analyzed using the MicroCal LLC DSC plug-in for Origin 7.0 software, provided with the instrument.

### Thioflavin T (ThT) fluorescence assay

DcpS protein samples at concentrations of 0.25 mg/ml, 0.5 mg/ml, and 1 mg/ml (in a 50 mM TRIS, 150 mM NaCl, and 0.2 mM TCEP pH 7.5 buffer) were incubated in the presence of Thioflavin T (Sigma-Aldrich) at a final concentration of 10 μM (0.0031886 mg/ml) in a total volume of 200 μl.

ThT assay in the presence of m^7^GDP was performed at the protein:ligand molar ratio 1:20, for 0.5 mg/ml DcpS concentration. DcpS was incubated with ligand for 30 min prior to ThT measurements.

Each sample was pipetted into a 96-well transparent plate (Greiner) and sealed with adhesive seals (Bio-Rad, Microseal ‘B’Adhesive Seals). ThT fluorescence was monitored using Synergy H1 (BioTek) microplate reader, at 487 nm emission and 440 nm excitation wavelengths. Measurements were performed at 37 °C with orbital agitation at 200 rpm over 10 days. All experiments were repeated at least three times, presented results are averaged from all measurements with standard deviations indicated.

### Formation of DcpS-derived amyloid fibrils

To form amyloid fibrils, DcpS at 0.5 mg/mL concentration (in a 50 mM TRIS, 150 mM NaCl, and 0.2 mM TCEP pH 7.5 buffer) in 200 μL volume were incubated at 37 °C over 10 days with continuous shaking in a microplate reader (Synergy H1, BioTek). Fibril formation was monitored in a parallel sample well where Thioflavin T was present (ThT fluorescence assay). After 10 days samples from wells without ThT were collected, and the morphology of the resulting DcpS-derived fibrils was examined by transmission electron microscopy (TEM). Samples of CeDcpS- and DcpS^INS1^-derived fibrils were sonicated (for 3 min in Elmasonic S30H ultrasound sonicator) prior to TEM imaging.

### Transmission electron microscopy

DcpS-derived fibrils, obtained as described above, were applied to a 400-mesh copper grid, coated with Formvar and carbon (Ted Pella), for 40 s and subsequently negatively stained with a 2% (w/v) water solution of uranyl acetate (Serva Electrophoresis GmbH ) for 25 s. The grids were dried at room temperature and the micrographs were collected using JEM 1400 transmission electron microscope (JEOL Co., Japan) equipped with 11-megapixel MORADA G2 TEM camera (EMSIS GmbH, Germany), operated at 80 kV.

### FT–IR measurements

Infrared spectra were collected using a Nicolet iS50 FTIR spectrometer (Thermo, USA) equipped with a single-reflection diamond ATR (Attenuated Total Reflectance) accessory and a DTGS detector. Centrifuged samples of DcpS aggregates collected from the plate reader (after 10 days incubation at 37 °C) were washed several times with H_2_O to remove buffer components. Liquid samples approximately 10 µL in volume were transferred onto the diamond surface of the ATR accessory and gently dried in situ. Subsequently, infrared spectra of the resulting films were recorded. Typically, 32 interferograms were collected at a resolution of 2 cm^−1^ to produce a single spectrum. The spectral data were processed with GRAMS software (Thermo).

### Fluorescence microscopy imaging

Liquid–liquid phase separation (LLPS) of protein samples was monitored using confocal fluorescence microscopy. Proteins were fluorescently labeled with ATTO 488 dye (Sigma-Aldrich) according to the manufacturer’s protocol. The DcpS protein was prepared at a final concentration of 1 mg/mL in 50 mM HEPES, 150 mM NaCl, pH 7.5, and incubated with ATTO 488 at room temperature for 1.5 h in a final volume of 50 μl. The molar ratio of protein to dye during labeling was 10:1. Unbound dye was removed using Amicon Ultra-15 centrifugal filters (10 kDa MWCO, Merck).

To induce droplet formation, labeled protein samples were incubated under the same buffer conditions in the presence of 5% or 10% PEG-4000 (as a macromolecular crowding agent). For imaging, 5–10 μl of each sample was applied to a glass slide and imaged either immediately or after the indicated incubation time. Fluorescence images were acquired using a Zeiss LSM 700 confocal microscope equipped with a 40 × oil immersion objective and processed using ZEISS ZEN 3.3 (Blue Edition) software.

To test the formation of ThT-positive aggregates within DcpS droplets, a mixture of CeDcpS (2 mg/ml) in 50 mM HEPES, 150 mM NaCl, pH 7.5, or in 50 mM TRIS, 150 mM NaCl, and 0.2 mM TCEP pH 7.5 buffer in the presence or absence of PEG-4000 was incubated with 10 µM ThT (in 30 µl final volume) at 37 °C. Aliquots of the prepared sample were transferred onto a glass slide (2 µl) at the start of incubation (t = 0 min) and after indicated time points, and microscopy images were taken with with Plan-Apo VC 100 × /1.40 oil immersion objective mounted on TE-2000E inverted microscope coupled with EZ-C1 Nikon confocal system. ThT was excited at 488 nm and fluorescence signal was collected using 515/30 nm filter. Images were processed with EZ-C1 software, FIJI and FigureJ.

### Fluorescence recovery after photobleaching (FRAP)

Fluorescence images of protein conjugated with Atto-488 were detected also using a Leica Stellaris 8 Falcon confocal system (Leica Microsystems) equipped with an inverted Leica DMI 8 microscope, a 100 × HC PL APO CS2 oil immersion objective lens (NA 1.4). Atto-488 was excited with 497 nm laser set to 0.5% of 85% of the maximum power of 80 MHz pulsed white light laser (1.8 mW). Note that laser intensity was relative so the exact laser power at the sample was not determined. Emission was detected using HyD S detector with spectral ranges set to 502–581 nm. Imaging settings in all measurements were kept constant. Images of 64 × 64 pixels were acquired with a scan speed of 400 Hz (bi-directional mode) at a zoom-factor of 48 and 2-times line-averaging. Hybrid detector was operated in analog detection mode with minimum gain 2.5. Altogether the settings resulted in a temporal resolution of 185 ms.

FRAP experiments were set up using the FRAP Wizard included in the Leica LAS X software (Leica Application Suite X). Labelled droplets of interest were randomly selected, and the focal plane was adjusted to the center. For qualitative experiments shown in Fig. 5B the outlined circle ROI1 (constant area) was photobleached with two laser line, 497 nm (at 100% of 85% of the maximum power of white light laser) and 405 nm (at 100%; 50 mW) and recovery of fluorescence monitored by scanning the whole droplet at low laser power (497 nm laser set to 0.5% of 85% of the maximum power of white light laser) in depicted intervals. The laser power used for intensity imaging did not significantly cause bleaching of the sample during the experiment. During FRAP experiments, ten frames were recorded before bleaching, four or two frames with a single bleaching for hDcpS and CeDcpS, respectively, followed by 120 frames (172 ms each) and 32 frames (500 ms each) after bleaching. A maximum of twenty FRAP measurements per protein were conducted. For analysis were six representative depicted. The same ROI surfaces were used to quantify fluorescence intensity values in bleached and unbleached areas. Recovery times, t1/2, tau, were calculated from time series by selecting single exponential recovery as curve fitting method using the in LAS X software (version 4.8.0.28989) and data were saved as individual reports and exported as csv files. Graphs were generated using Origin Pro8 (Origin Lab) software. Mobile fraction was calculated according to formula M_f_ = (F−F_bleach_)/(F_pre_−F_bleach_) where F is the fluorescence intensity at the plateau after recovery, F_bleach_ is the intensity immediately after bleaching, and F_pre_ is the pre-bleach intensity. Representative images from the FRAP experiment were compiled with FIJI/ImageJ (ver. 2.14.0/1.54f.; https://imagej.net/software/fiji/).

## Supplementary Information

Below is the link to the electronic supplementary material.

Supplementary Material 1.

## Data Availability

All data generated or analyzed during this study are included in this published article and its Supplementary information files.
