# Supplementary material for: Liquid–liquid phase separation and the formation of amyloid fibrils from DcpS scavenger enzymes
Source: Sci Rep. 2026 May 27;16:24207. doi: 10.1038/s41598-026-55504-x (PMC13444105; doi:10.1038/s41598-026-55504-x)
Supplement: Supplementary file 1 [file 41598_2026_55504_MOESM1_ESM.pdf]

SUPPLEMENTARY INFORMATION

Liquid-Liquid Phase Separation and the formation of amyloid fibrils from DcpS scavenger enzymes.

Aleksandra Ferenc-Mrozek[1], Maria Winiewska-Szajewska[1,2], Hanna Nieznańska[3], Anna Anielska-Mazur[2], Marek Warzecha[1], Wojciech Dzwolak[4]* and Maciej Łukaszewicz[1]*

[1] Division of Biophysics, Faculty of Physics, University of Warsaw, Pasteura 5, 02-093 Warsaw, Poland

[2] Institute of Biochemistry and Biophysics PAS, Pawinskiego 5a, 02-106, Warsaw, Poland

[3] Laboratory of Electron Microscopy, Nencki Institute of Experimental Biology PAS, Pasteura 3, 02-093 Warsaw, Poland

[4] Faculty of Chemistry, Biological and Chemical Research Centre, University of Warsaw, Pasteura 1, 02-093 Warsaw, Poland

*Corresponding authors: Maciej Lukaszewicz (Maciej.Lukaszewicz@fuw.edu.pl), Wojciech Dzwolak (wdzwolak@chem.uw.edu.pl)

Supplementary Table 1. Aggregation prone regions (APR) predicted by WALTZ algorithm for human DcpS, mutant human DcpS with 15aa insertion and *C. elegans* DcpS. APR segments are numbered according to appearance in the amino acid sequence of DcpS.

APR	**Human DcpS**	**Human DcpS ^{INS15}**	***C.elegans* DcpS**
segment no.1	^{173}IQWVYNI^{179}	^{173}IQWVYNI^{179}	^{147}LNWVYN^{152}
segment no. 2	^{199}GFVLIP^{204}	^{199}GFVLIP^{204}	^{187}LENLYVLAI^{195}
		^{218}WNVLIS^{223}	

A **B**

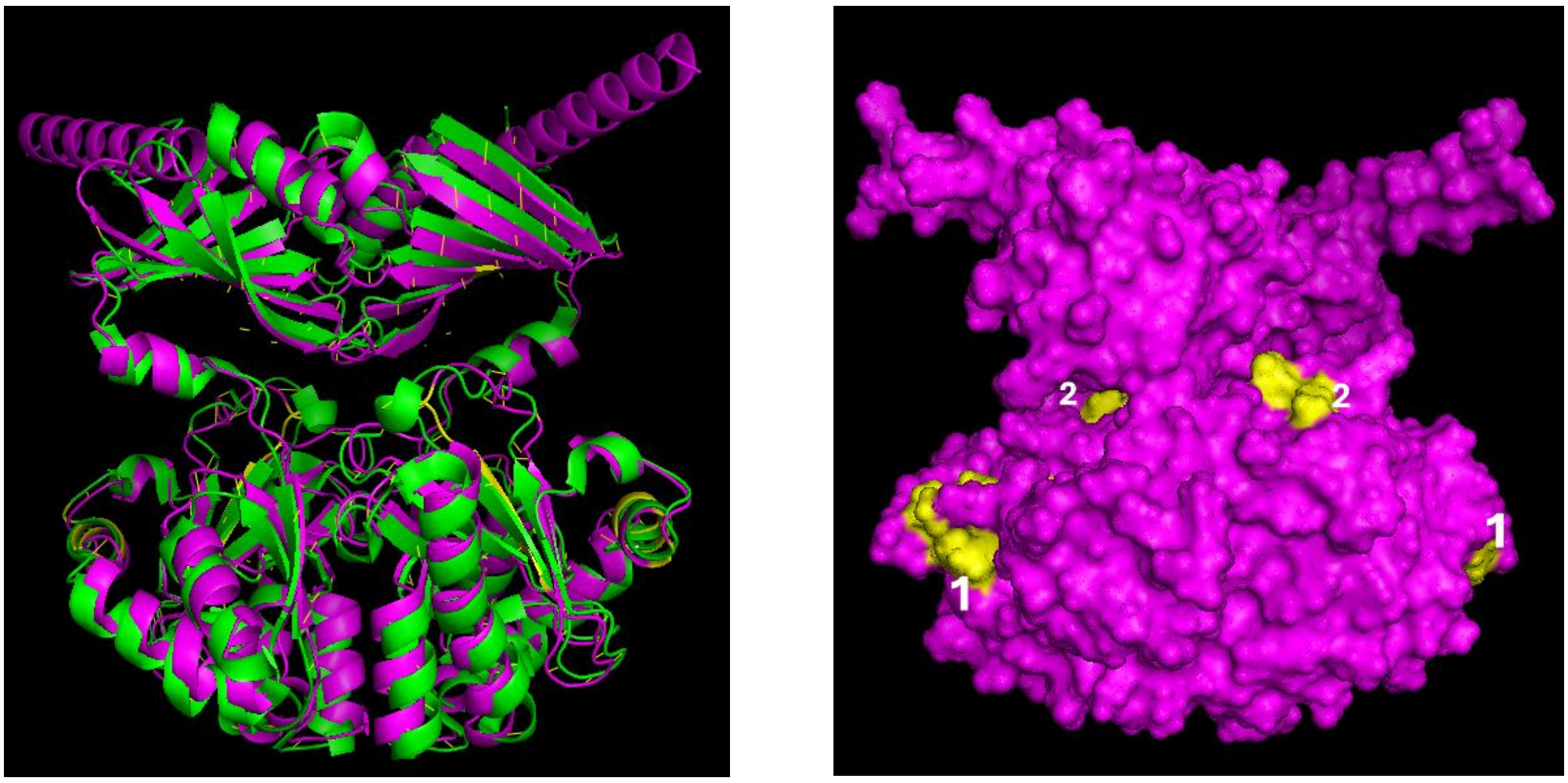

Supplementary Figure 1. (A) Comparison of AlphaFold prediction model of *C .elegans* DcpS structure (**magenta**) with human DcpS structure (PDB:1XML) (**green**). *C. elegans* DcpS structure was predicted using AlphaFold 2 (Jumper *et.al.,* 2021). **(B)** Localization of the predicted APR segments (no.1 and no.2) (**yellow**) within the predicted dimeric structure of *C. elegans* DcpS.

A.

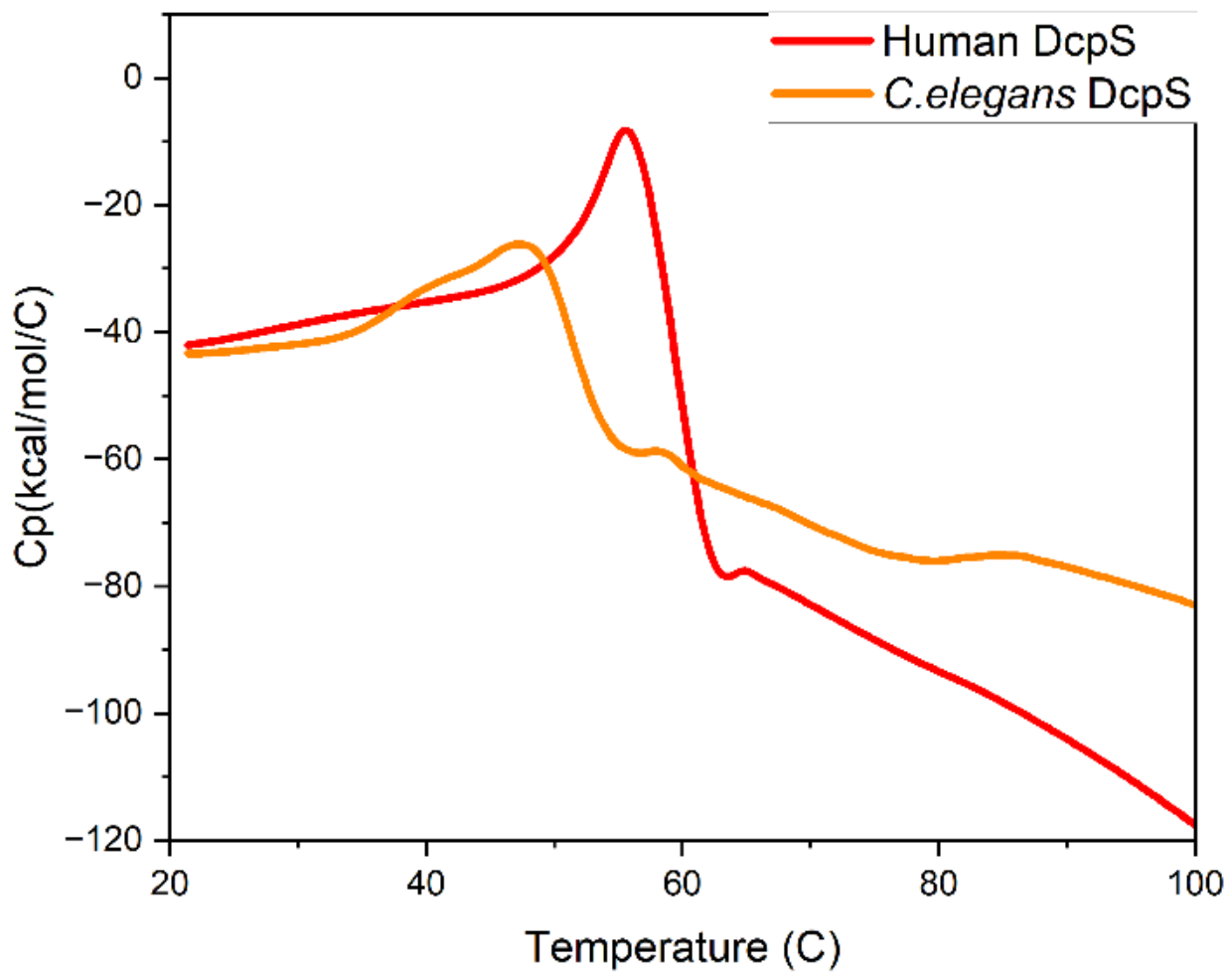

B.

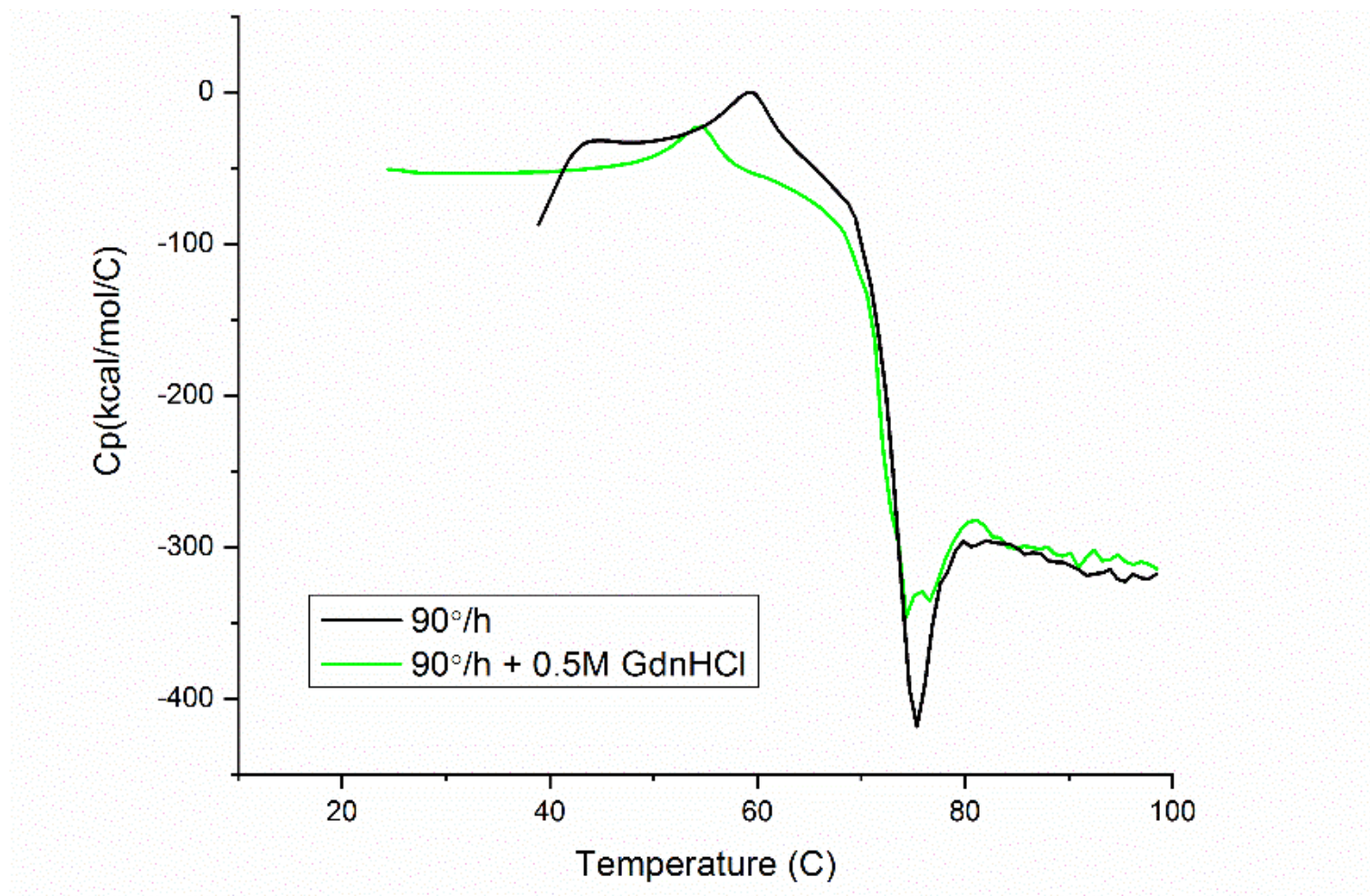

Supplementary Figure 2.

A) DSC heat capacity profiles demonstrating changes in the apparent molar heat capacity of aggregating DcpS proteins from humans and *C. elegans*. A positive peak is observed, indicating an endothermic transition at 57°C for human DcpS and 48°C for *C. elegans* DcpS, preceding another endothermic transition. Experiments were performed at 0.7 mg/ml protein concentration in 50 mM TRIS, 150 mM NaCl, 0.2 mM TCEP, pH 7.5, at a scanning rate 0.5°C/min.

B) DSC heat capacity profiles for human DcpS at a scanning rate 1.5°C/min (90°C/h, black line) and in the presence of 0.5M GdnHCl. As it is seen, the endothermic unfolding and exothermic aggregation transitions were successfully resolved by employing an increased scanning rate (90 °C/h). Unlike the subsequent aggregation, the unfolding process is relatively rapid and diffusion-independent. Consequently, higher scanning rates lead to only a modest upward temperature shift in the endothermic peak, whereas the shift for the exothermic aggregation is significantly more pronounced (Dzwolak *et.al.* 2003). Combining the high scan rate with a moderate concentration of the chaotropic denaturant GdnHCl further improved peak resolution by selectively shifting the unfolding transition toward lower temperatures.

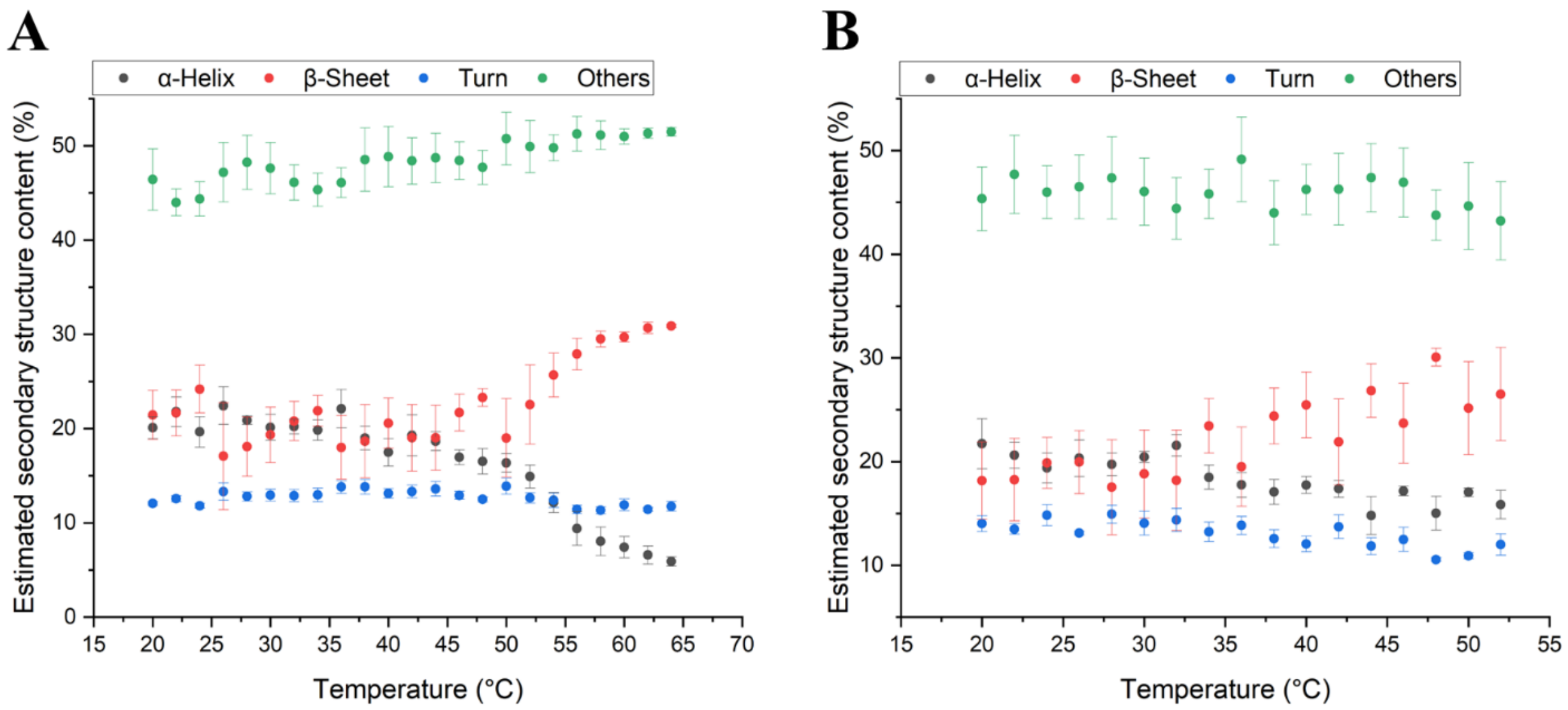

Supplementary Figure 3. Estimated secondary structure content of human **(A)** and *C. elegans* **(B)** DcpS by BeStSel server according to CD measurements at temperatures ranging from 20°C to 64°C for human DcpS and form 20°C to 52°C for *C. elegans* DcpS. The percentages of α-helix and β-sheet are represented by filled black and red circles, respectively. The data presented correspond to four independent experiments, and the average secondary structures content (± standard deviation) is shown.

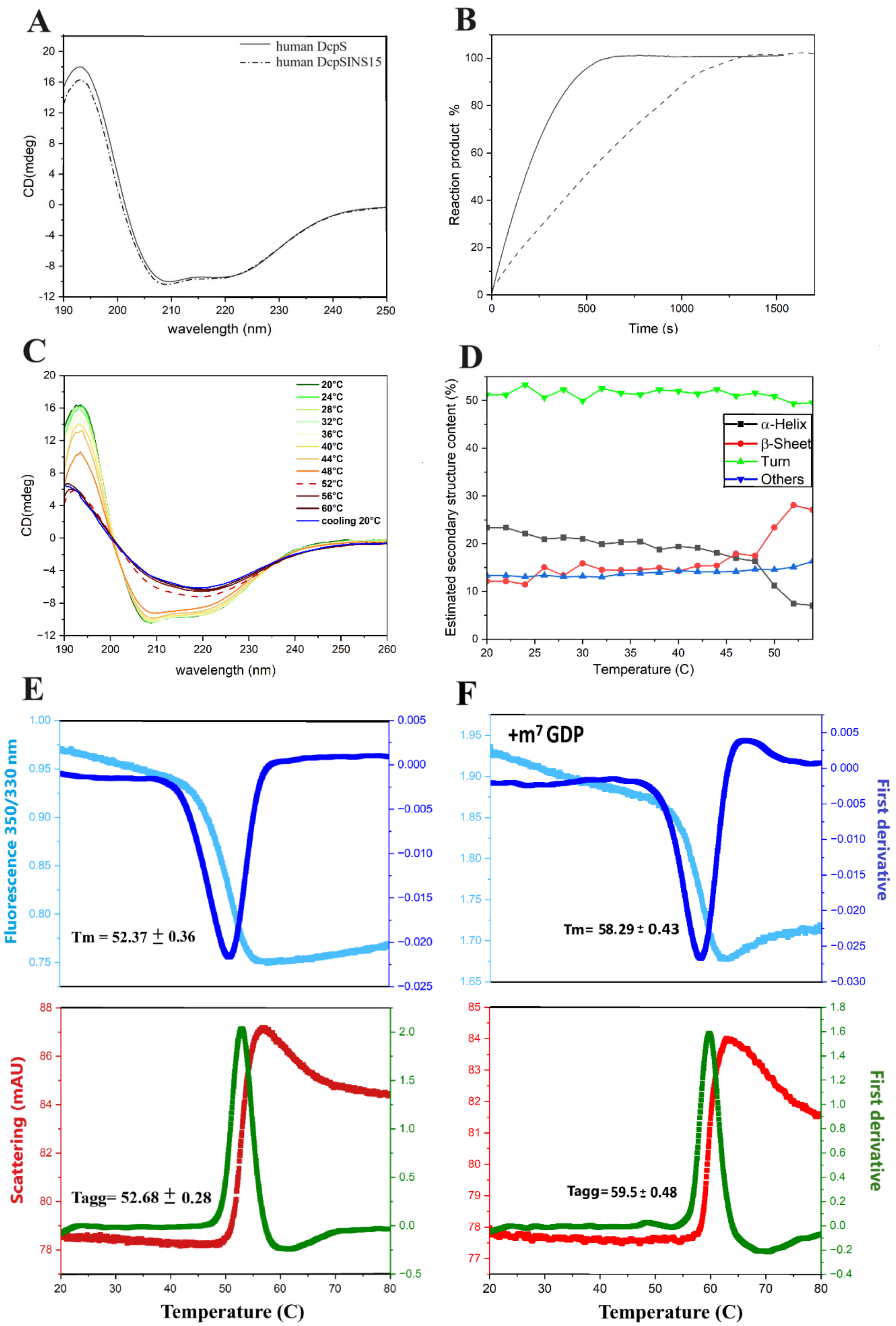

Supplementary Figure 4. Biophysical properties of human mutant DcpS^{INS15} protein.

Supplementary Figure 4. Biophysical properties of human mutant DcpS^{INS15} protein. (continued)

A. Comparative far-UV CD spectra of human DcpS and its insertional mutant DcpS^{INS15}

B. Kinetics of cap hydrolysis catalyzed by human DcpS (smooth line for WT DcpS and dashed line for DcpS^{INS15}). Reaction progress curves were obtained for the 10 μM m^{7}GpppG dinucleotide substrate in the presence of 30 nM of the indicated enzyme.

C. Overlaid smoothed far-UV CD spectra of DcpS^{INS15} obtained at a temperatures ranging from 20°C to 60°C (what is 8°C above the estimated with nanoDSF melting temperature Tm, dashed line). Green line is for the spectrum obtained at 20°C, followed by the series of rainbow-colored lines corresponding to plots obtained at 2°C intervals. In dark blue is shown spectrum obtained after cooling at 20°C for 12 hours the DcpS^{INS15} protein sample heated to 52°C. Spectra were acquired at the heating rate 0.5°C/min.

D. Estimated secondary structure content by BeStSel server according to CD measurements at temperatures ranging from 20°C to 60°C. The percentages of α-helix and β-sheet are represented by filled black squares and red circles, respectively.

E and **F.** Thermal stability of DcpS^{INS15} in the absence (**E**) or presence of m^{7}GDP nucleotide ligand (**F**), measured by nanoDSF (upper panels). Presented thermograms display the first derivative (dark blue) of fluorescence signal intensity ratio at 350nm and 330nm emission wavelength (light blue) under thermal unfolding in the temperature range 20°C – 80°C. The temperature at which melting transition (Tm) occur is shown.

Bottom panels in **E** and **F** shows measured scattering profiles for DcpS^{INS15} (red line) in temperature range 20°C – 80°C (using NanoDSF Prometheus NT.48 with back-reflection optics). Green line corresponds to the first derivative of a scattering profile, and calculated aggregation temperature is shown. For nanoDSF thermal stability and scattering measurments the heating rate was 0.5°C/min.

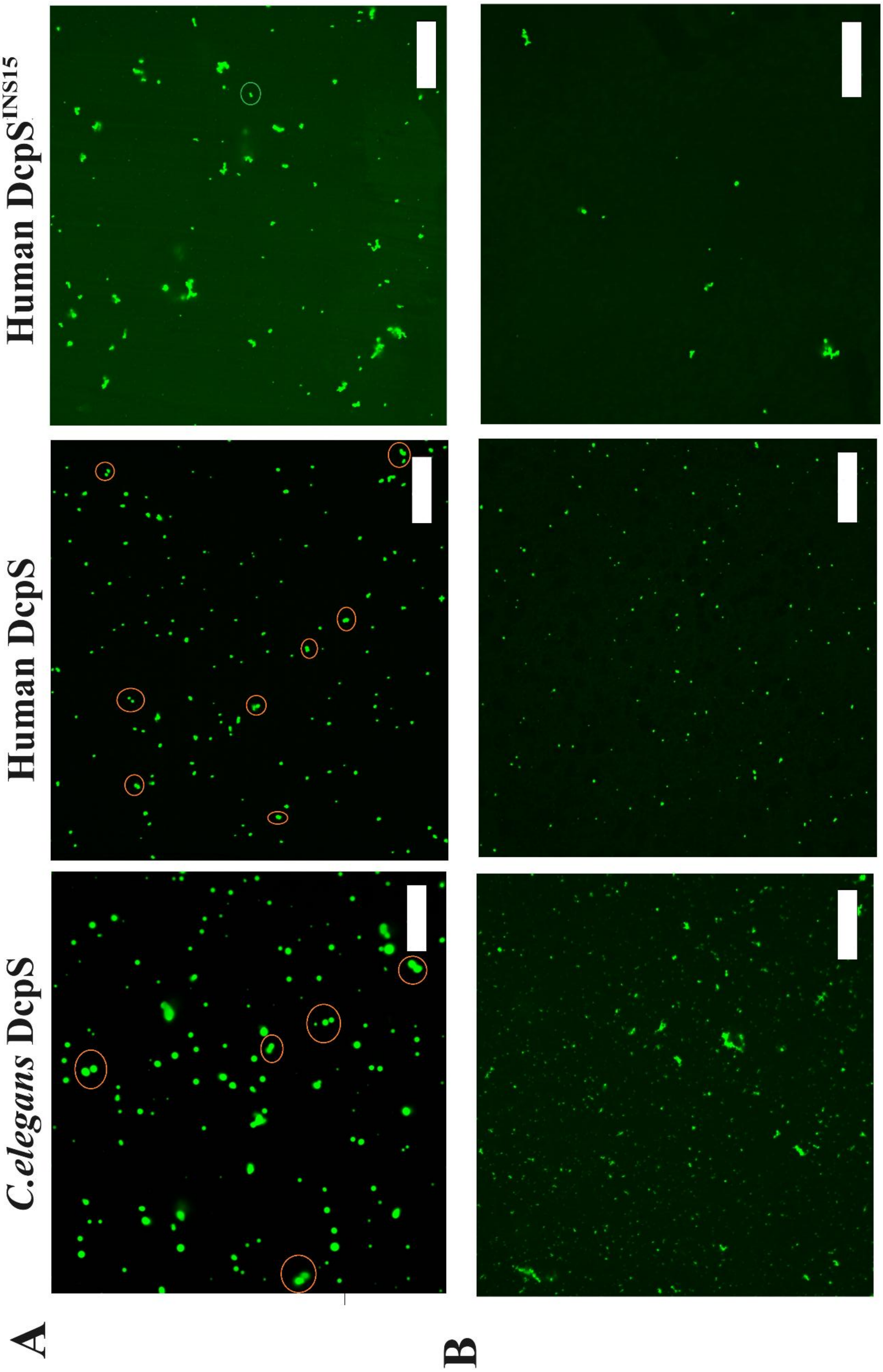

Supplementary Figure 5.

Supplementary Figure 5. Confocal microscopic images of DcpS droplets. (continued)

(**A)** 5 hours incubation in 20°C in presence of 5% PEG 4000 result in fusion event for *C. elegans* DcpS and human DcpS, while mutant $DcpS^{INS15}$ presents irregular probably aggregated structures. Representation of potential fusion events between DcpS droplets are marked by circles.

(**B)** After 30 minutes incubation in 37°C, small potential aggregates appeared for *C. elegans* DcpS and human mutant $DcpS^{INS15}$, and human WT DcpS droplets were smaller with weaker tendency to fusion than in 20°C. Data obtained in a buffer containing 50mM HEPES, 150mM NaCl pH 7.5. Scale bars 20µm.

A.

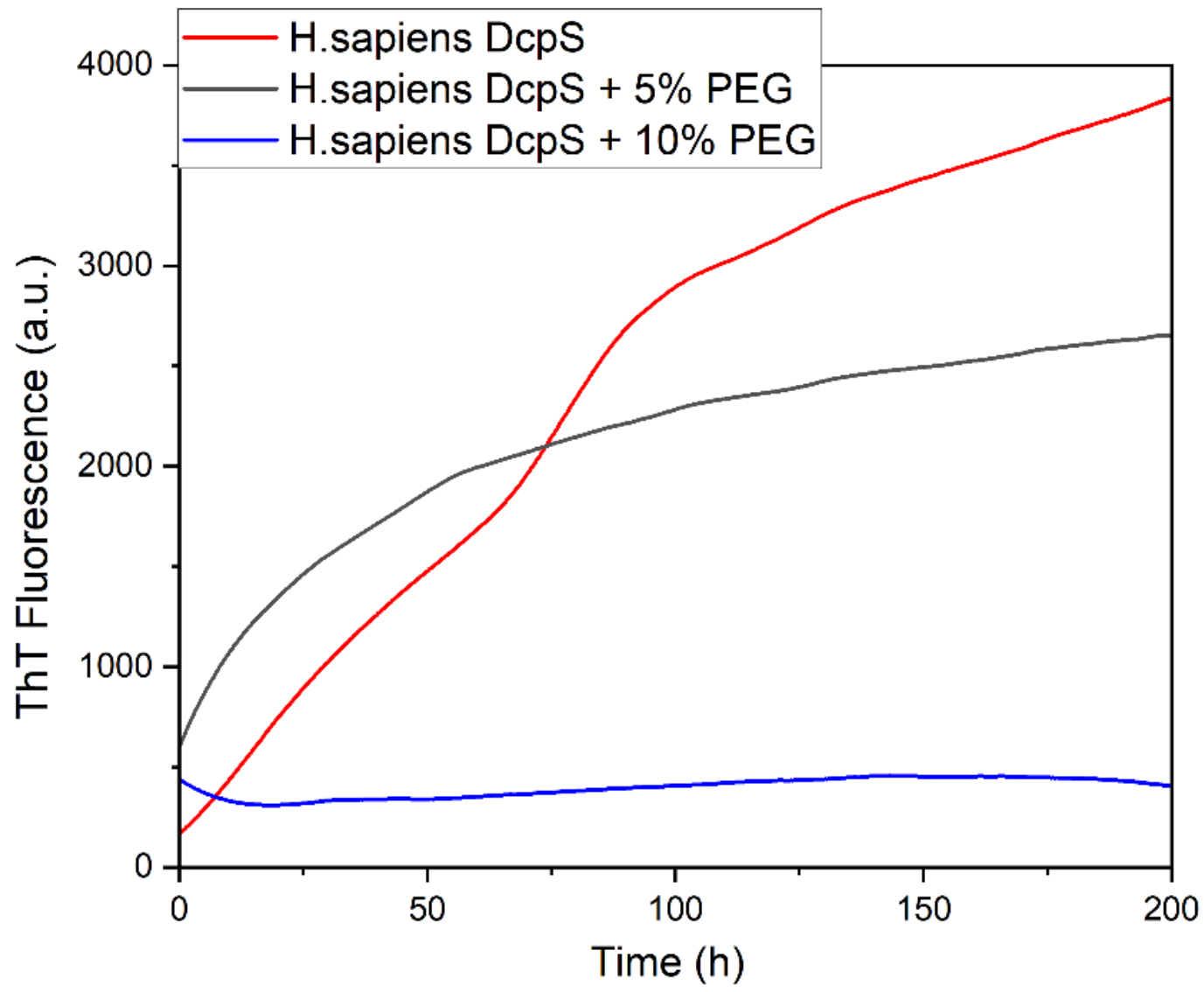

B.

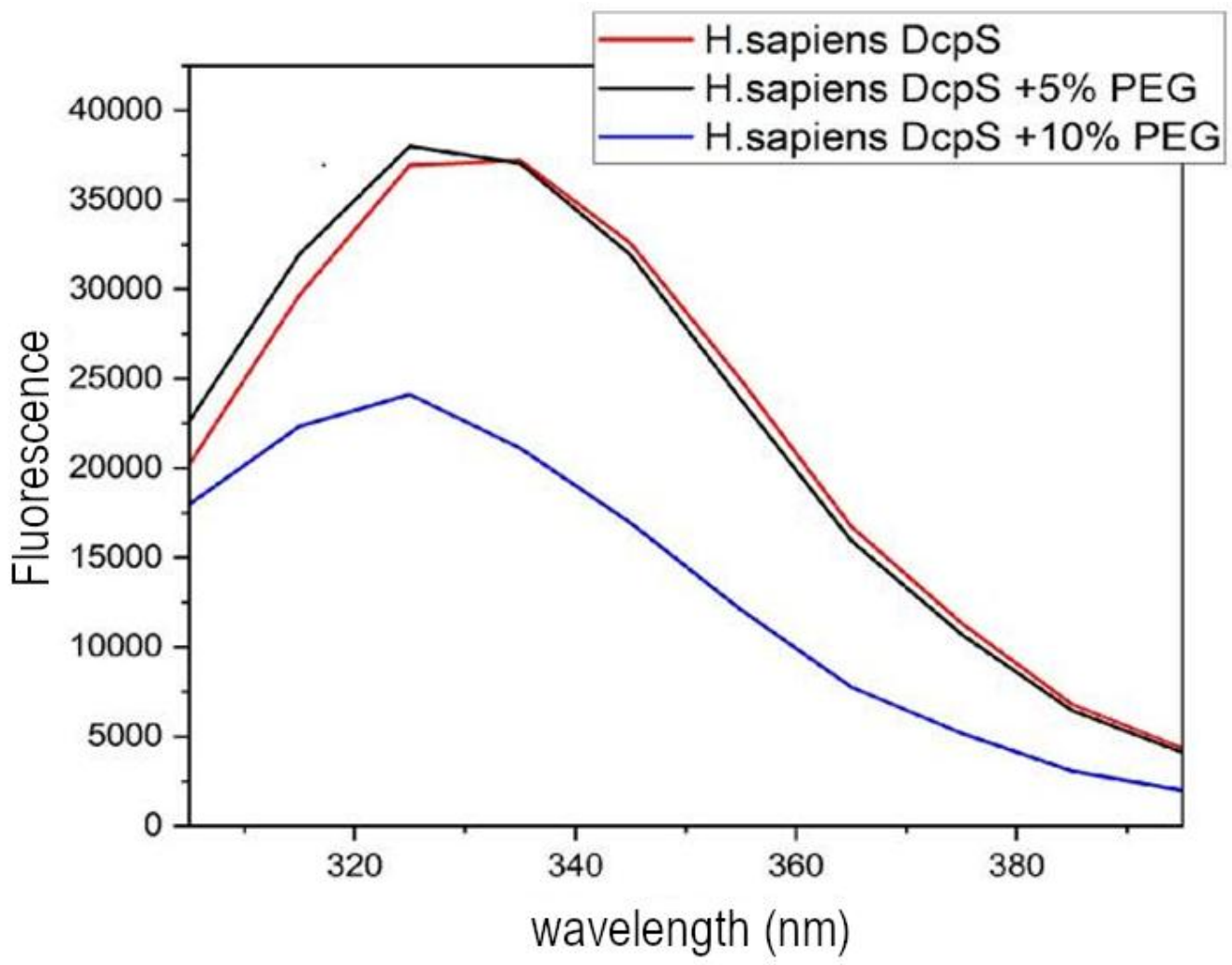

Supplementary Figure 6. A) Thioflavin T fluorescence intensity of human DcpS under molecular crowding conditions: without PEG 4000 (red line - control), with 5% PEG 4000 (grey line) and 10% PEG 4000 (blue line). Protein concentration – 0.5mg/ml.

B) Comparison of the fluorescence spectra of human DcpS (excitation wavelength 295nm) in the presence of 5% PEG 4000 (black line) and 10% PEG 4000 (blue line). As can be seen, there is a significant decrease in the intrinsic fluorescence signal of the protein at 10% PEG, but no apparent change at 5% PEG compared to non-crowding conditions. This suggest that higher PEG induce structural changes in DcpS (with changes in the local environment of tryptophan – two such residues are present in HsDcpS and three in CeDcpS, and one of those is involved recognition of a substrate via stacking interaction in both species, Wypijewska del Nogal et.al.). Similar observations (intrinsic fluorescence quenching) were reported at higher concentrations of PEG 8K and PEG 20K as a crowding agent for proryl-tRNA synthase ProRS (Liebau et.al., 2024)

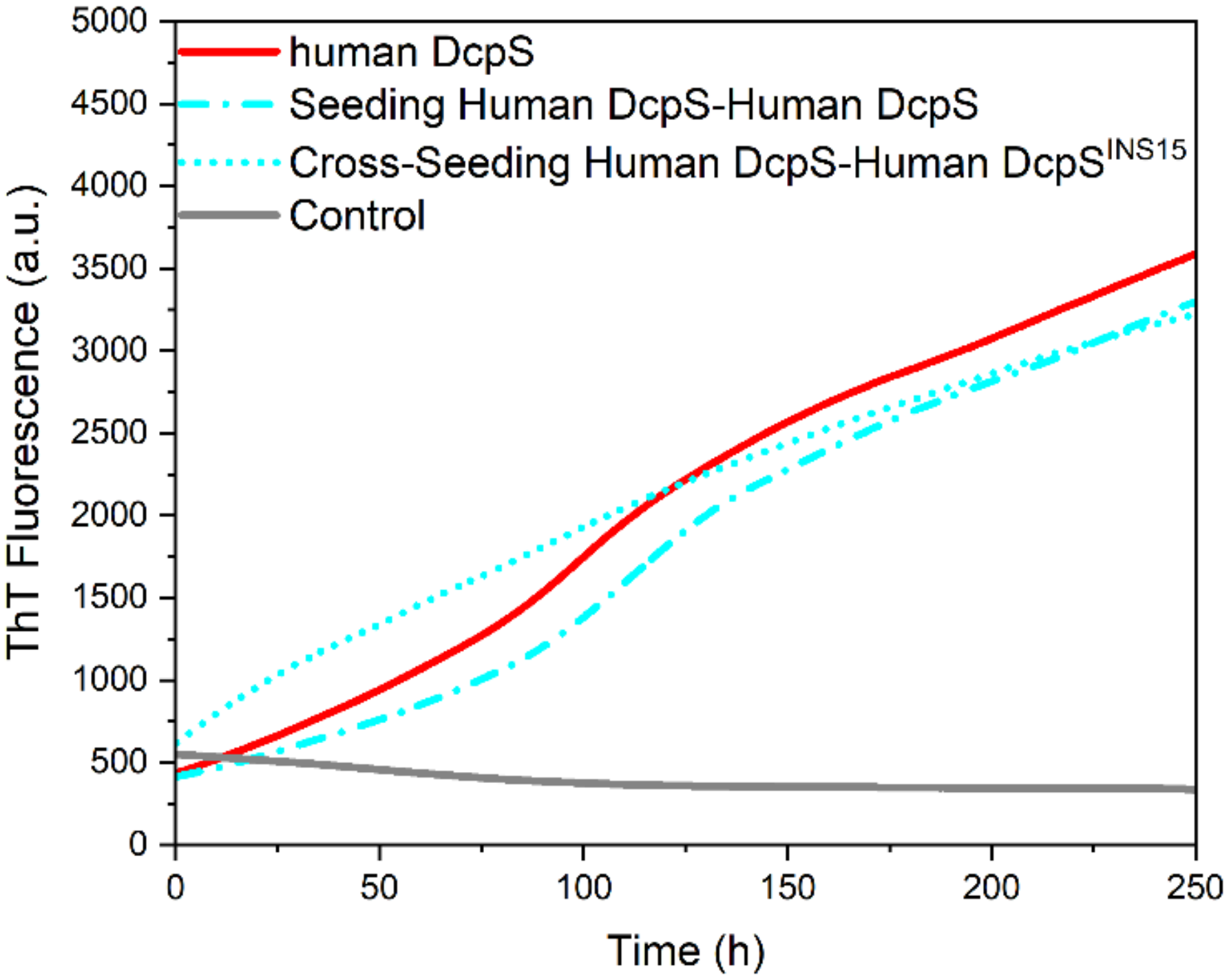

Supplementary Figure 7. Aggregation kinetics by ThT assay of human DcpS in the presence or absence of homogenic or heterogenic preformed DcpS seeds. Samples of purified DcpS (0.5mg/ml) were incubated alone (solid red line) or with sonicated fibrillar aggregates of wild-type human DcpS (dash-dot cyan line) or its mutant - DcpS^{INS15} (dotted cyan line), at seeds to DcpS ratio 20:1. Grey solid line correspond to control (in 50mM TRIS, 150mM NaCl, 0.2mM TCEP, pH 7.5 buffer) with sonicated human DcpS fibrillar aggregates to make a sure that seeds do not generate false positive signal at concentrations used in seeding experiments.

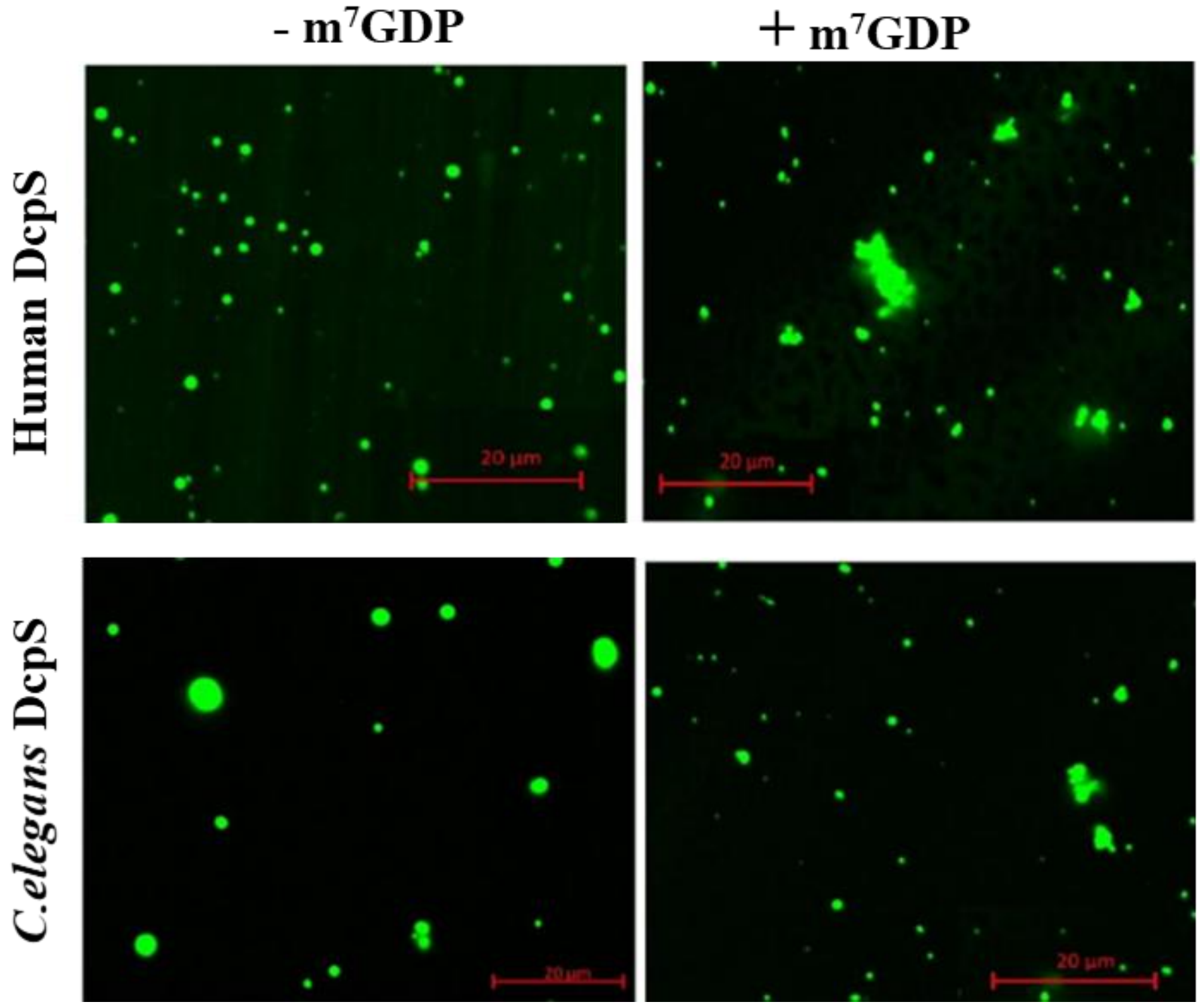

Supplementary Figure 8. Confocal microscopic images of human and *C. elegans* DcpS droplets in the presence of m^{7}GDP

Images of ATTO488-labeled human and *C. elegans* DcpS in the absence and presence of m^{7}GDP. m^{7}GDP ligand was added to protein sample at 10-times molar excess and incubated for 30 min at 20°C before PEG4000 addition to 5% final concentration. Data obtained 50mM HEPES, 150mM NaCl pH 7.5. Scale bars 20µm.

A.

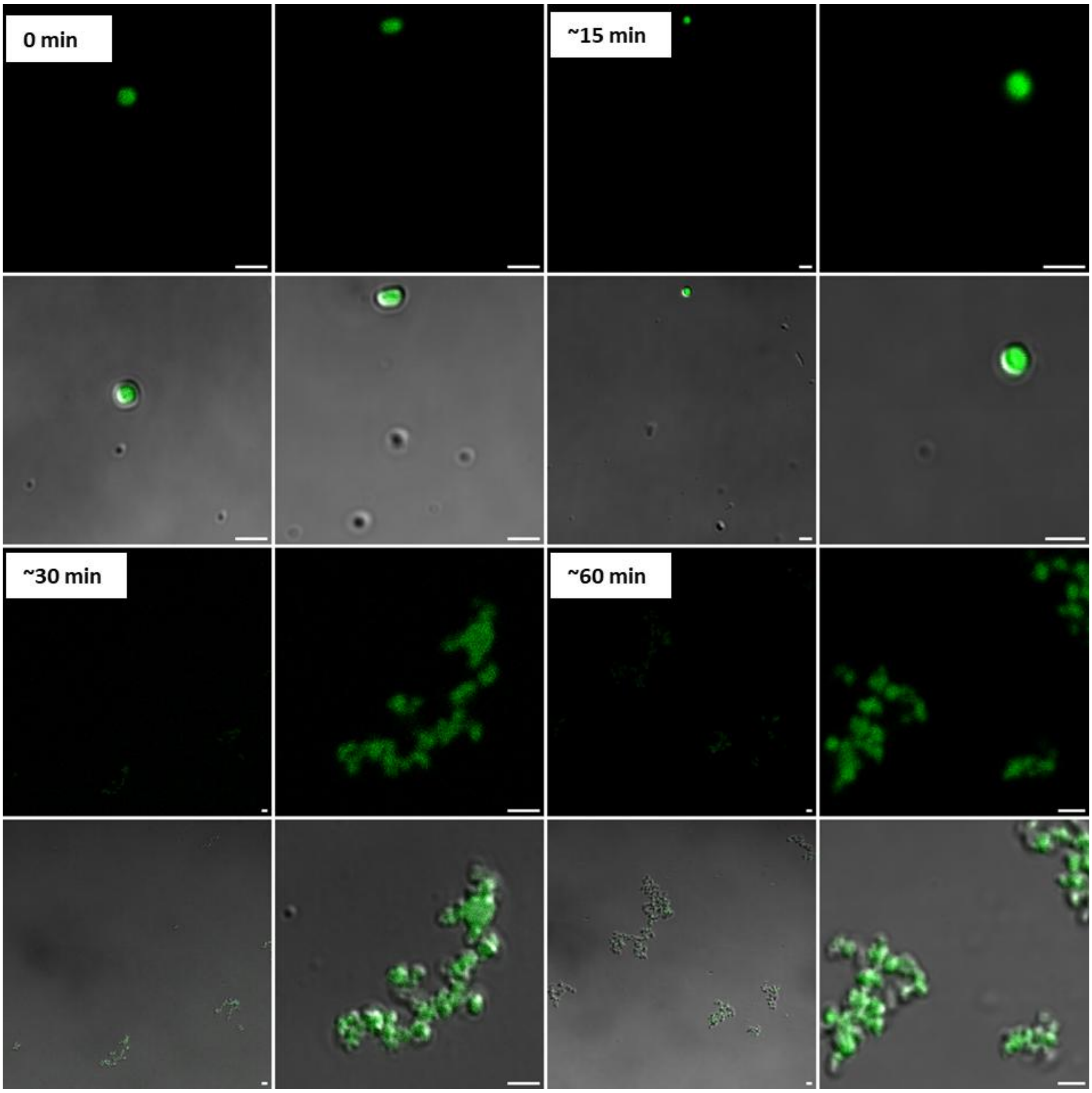

Supplementary Figure 9 A. Confocal microscopic images of ThT-positive aggregates within CeDcpS-formed droplets in the presence of PEG-4000: **(A)** in a 50 mM HEPES/150 mM NaCl buffer at pH 7.5, and **(B,** shown below**)** in a 50 mM Tris/150 mM NaCl/2 mM TCEP buffer at pH 7.5 (that was used in the DcpS ThT kinetics fibrilization assays). ThT was present at 10 μM final concentration. Merged images of ThT fluorescence and the bright-field channel (upper panel), and the ThT fluorescence alone (bottom panel) are shown for the indicated time points. ThT-positive droplets are visible next to droplets lacking fluorescence already at the start of the experiment (0 min), and at ~30 minutes of the incubation larger ThT-positive aggregates are formed. Incubation was at 37°C, with no shaking. Scale bar correspond to 2 μm.

B.

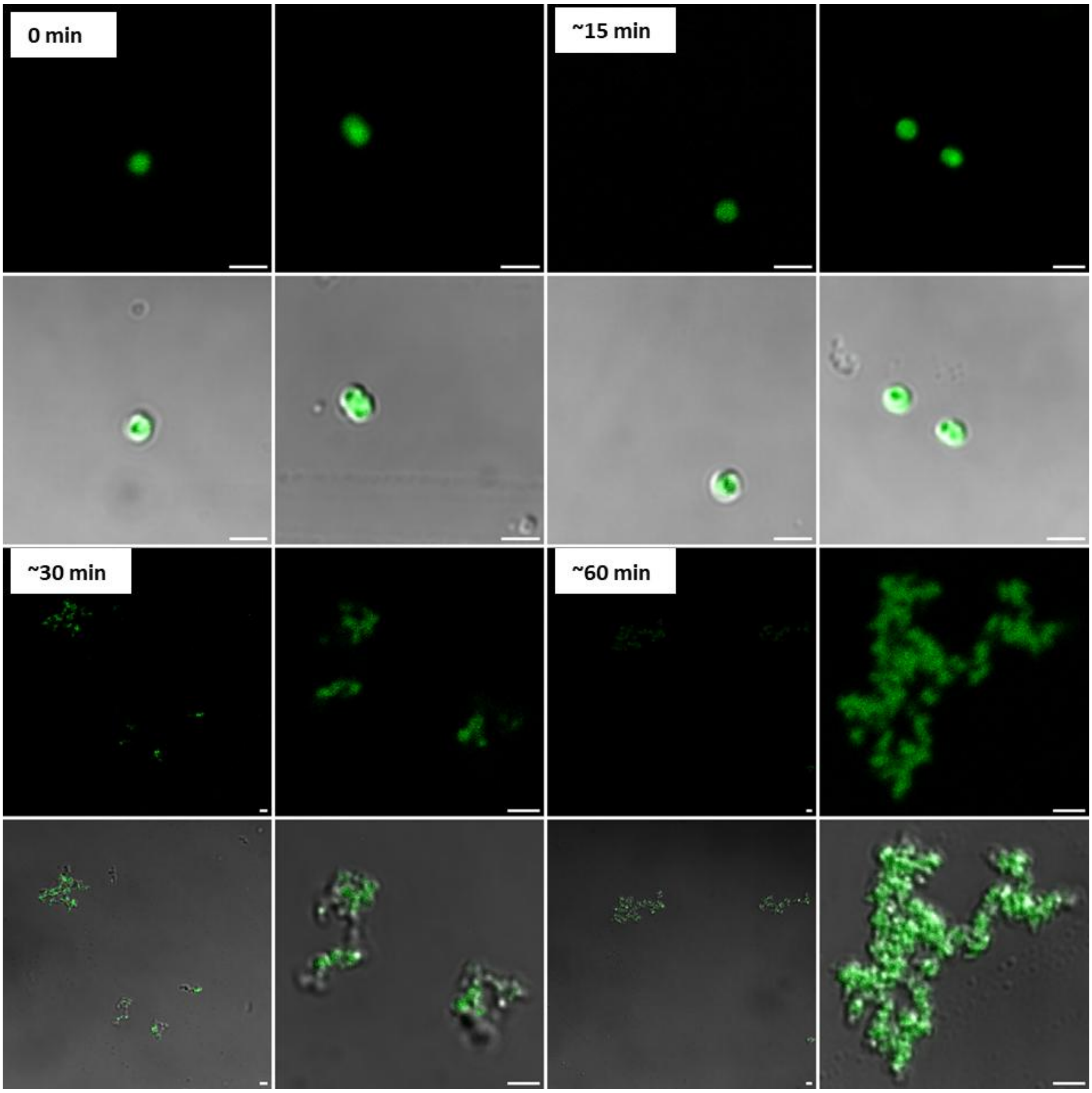

Supplementary Figure 9 B. Confocal microscopic images of ThT-positive aggregates within CeDcpS-formed droplets in the presence of PEG-4000 **(B)** in a 50 mM Tris/150 mM NaCl/2 mM TCEP buffer at pH 7.5 (*detailed description above*).

A.

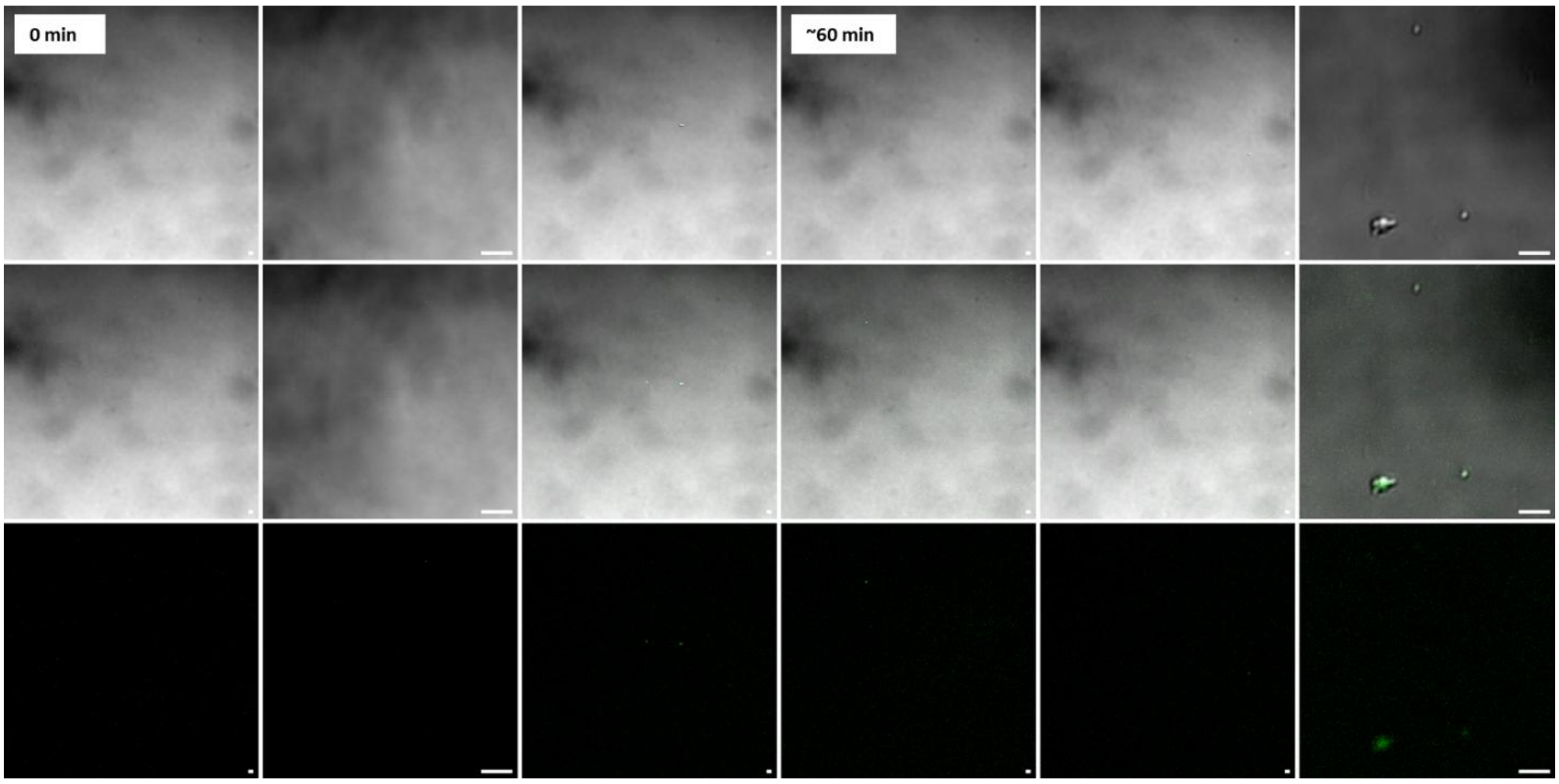

B.

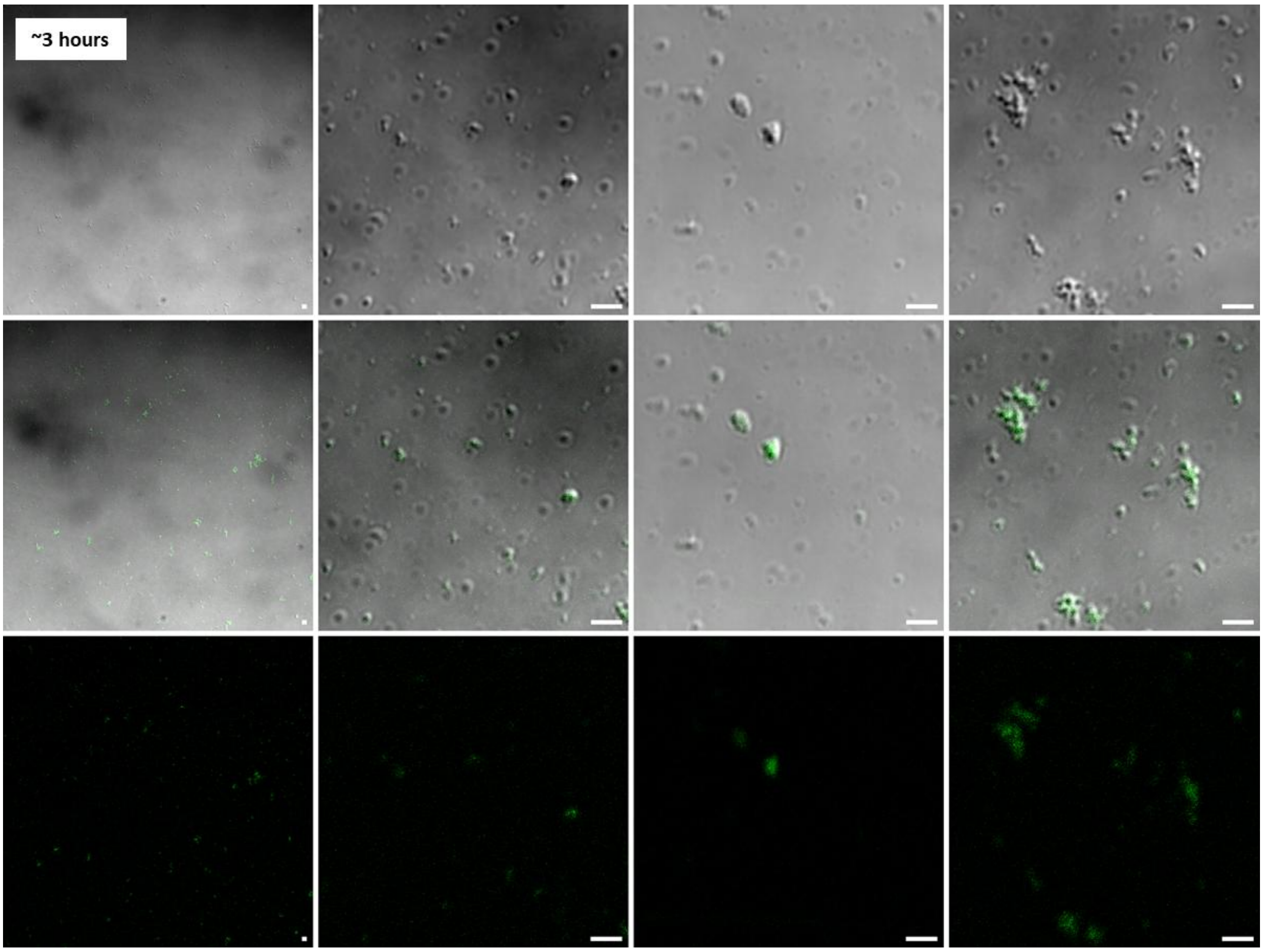

Supplementary Figure 10. Microscopic analysis of CeDcpS without PEG molecular crowder.

Supplementary Figure 10. Microscopic analysis of CeDcpS without PEG molecular crowder. (*continued*)

Images were acquired for CeDcpS in the presence of ThT-dye (10 µM final concentration) and in 50 mM Tris/ 150 mM NaCl/ 2 mM TCEP buffer at pH 7.5 buffer, without PEG-4000. Bright-field (upper panel), the corresponding merged images of ThT fluorescence and the bright-field channel (middle panel), and the ThT fluorescence alone images (bottom panel) are shown. (**A**) Representative images taken at the start (0min) and after 60 minutes of incubation at 37°C. As it is seen, no apparent droplets are detected up to around 60 min of incubation (compared to rapid droplets formation in the presence of PEG crowding agent (Supplementary Figure 9B). (**B**) Without PEG, the visible droplets and ThT-positive aggregates appears later, after ~3 hours of incubation. Scale bar correspond to 2 µm.

Supplementary references

Dzwolak W., Ravindra R., Lendermann J., Winter R. (2003) Aggregation of Bovine Insulin Probed by DSC/PPC Calorimetry and FTIR Spectroscopy. Biochemistry 42 (38), 11347-11355 DOI: 10.1021/bi034879h

Jumper, J., Evans, R., Pritzel, A., Green, T., Figurnov, M., Ronneberger, O., Tunyasuvunakool, K., Bates, R., Žídek, A., Potapenko, A., Bridgland, A., Meyer, C., Kohl, S. A. A., Ballard, A. J., Cowie, A., Romera-Paredes, B., Nikolov, S., Jain, R., Adler, J., Back, T., … Hassabis, D. (2021). Highly accurate protein structure prediction with AlphaFold. *Nature*, *596*(7873), 583–589. https://doi.org/10.1038/s41586-021-03819-2

Liebau, J., Laatsch, B. F., Rusnak, J., Gunderson, K., Finke, B., Bargender, K., Narkiewicz-Jodko, A., Weeks, K., Williams, M. T., Shulgina, I., Musier-Forsyth, K., Bhattacharyya, S., & Hati, S. (2024). Polyethylene Glycol Impacts Conformation and Dynamics of Escherichia coli Prolyl-tRNA Synthetase Via Crowding and Confinement Effects. Biochemistry, 63(13), 1621–1635. https://doi.org/10.1021/acs.biochem.3c00719

Wypijewska del Nogal, A., Surleac, M. D., Kowalska, J., Lukaszewicz, M., Jemielity, J., Bisaillon, M., Darzynkiewicz, E., Milac, A. L., & Bojarska, E. (2013). Analysis of decapping scavenger cap complex using modified cap analogs reveals molecular determinants for efficient cap binding. The FEBS journal, 280(24), 6508–6527. https://doi.org/10.1111/febs.12553
